# Hyperbaric Oxygen Therapy Can Improve Post Concussion Syndrome Years after Mild Traumatic Brain Injury - Randomized Prospective Trial

**DOI:** 10.1371/journal.pone.0079995

**Published:** 2013-11-15

**Authors:** Rahav Boussi-Gross, Haim Golan, Gregori Fishlev, Yair Bechor, Olga Volkov, Jacob Bergan, Mony Friedman, Dan Hoofien, Nathan Shlamkovitch, Eshel Ben-Jacob, Shai Efrati

**Affiliations:** 1 The Institute of Hyperbaric Medicine, Assaf Harofeh Medical Center, Zerifin, Israel; 2 Research and Development Unit, Assaf Harofeh Medical Center, Zerifin, Israel; 3 Sackler School of Medicine, Tel-Aviv University, Tel-Aviv, Israel; 4 Nuclear Medicine institute, Assaf Harofeh Medical Center, Zerifin, Israel; 5 The Raymond and Beverly Sackler Faculty of Exact Sciences, School of Physics and Astronomy, Tel-Aviv University, Tel-Aviv, Israel; 6 Department of Psychology, The Hebrew University of Jerusalem, Jerusalem, Israel; 7 The National Institute for the Rehabilitation of the Brain Injured, Tel-Aviv, Israel; 8 Otolaryngology, Head & Neck Surgery, Assaf-Harofeh Medical Center, Zerifin, Israel; 9 Center for Theoretical Biological Physics, Rice University, Houston, Texas, United States of America; 10 Sagol School of Neuroscience, Tel-Aviv University, Tel-Aviv, Israel; St Michael's Hospital, University of Toronto, Canada

## Abstract

**Background:**

Traumatic brain injury (TBI) is the leading cause of death and disability in the US. Approximately 70-90% of the TBI cases are classified as mild, and up to 25% of them will not recover and suffer chronic neurocognitive impairments. The main pathology in these cases involves diffuse brain injuries, which are hard to detect by anatomical imaging yet noticeable in metabolic imaging. The current study tested the effectiveness of Hyperbaric Oxygen Therapy (HBOT) in improving brain function and quality of life in mTBI patients suffering chronic neurocognitive impairments.

**Methods and Findings:**

The trial population included 56 mTBI patients 1–5 years after injury with prolonged post-concussion syndrome (PCS). The HBOT effect was evaluated by means of prospective, randomized, crossover controlled trial: the patients were randomly assigned to treated or crossover groups. Patients in the treated group were evaluated at baseline and following 40 HBOT sessions; patients in the crossover group were evaluated three times: at baseline, following a 2-month control period of no treatment, and following subsequent 2-months of 40 HBOT sessions. The HBOT protocol included 40 treatment sessions (5 days/week), 60 minutes each, with 100% oxygen at 1.5 ATA. “Mindstreams” was used for cognitive evaluations, quality of life (QOL) was evaluated by the EQ-5D, and changes in brain activity were assessed by SPECT imaging. Significant improvements were demonstrated in cognitive function and QOL in both groups following HBOT but no significant improvement was observed following the control period. SPECT imaging revealed elevated brain activity in good agreement with the cognitive improvements.

**Conclusions:**

HBOT can induce neuroplasticity leading to repair of chronically impaired brain functions and improved quality of life in mTBI patients with prolonged PCS at late chronic stage.

**Trial Registration:**

ClinicalTrials.gov

NCT00715052

## Introduction

Traumatic brain injury (TBI) and stroke are the major causes of brain damage. Every year, close to two million people in the US suffer TBI, which is the leading cause of death and disability among the general population. Stroke affects almost a million people and is the leading cause of inability to maintain independent life among adults [1], [2]. There is no effective treatment/metabolic intervention in the daily clinical practice for post TBI and stroke patients with chronic neurological dysfunction. Intensive therapy and rehabilitation programs are considered essential for maximizing quality of life but are often just partially successful. Clearly, new methods for brain repair should be examined in order to provide sustained relief to brain damage patients. Recent studies reported that hyperbaric oxygen treatment (HBOT) can induce neuroplasticity leading to significant neurological improvement in post-stroke patients at the convalescent stage and at late chronic stages, months to years after the acute event [3], [4].

### Definitions and classifications

Traumatic brain injury is defined as damage to the brain resulting from external mechanical force, such as rapid acceleration or deceleration, impact, blast waves, or penetration by a projectile. Consequently to the injury, brain function is temporarily or permanently impaired and structural damage may or may not be detectable with current imaging technology. TBI is usually classified based on severity, anatomical features of the injury, and the cause of the injury. The severity is assessed according to the loss of consciousness (LOC) duration, the post-traumatic amnesia (PTA), and the Glasgow coma scale (GCS) grading of the level of consciousness. Approximately (70–90%) of the TBI in the US are classified as mild TBI (mTBI) or concussion – LOC duration of 0–30 minutes, PTA duration of less than a day and GCS grade of 13–15. Post concussion syndrome (PCS) is a set of symptoms succeeding mTBI in most patients. The PCS symptoms include headache, dizziness, neuropsychiatric symptoms, and cognitive impairments [5], [6]. In most patients, PCS may continue for weeks or months, and up to 25% of the patients may experience prolonged PCS (PPCS) in which the symptoms last for over six months [7], [8], [9], [10], [11], [12]. Such individuals are at high risk for emotional and cognitive dysfunction, culminating in inability to carry out ordinary daily activities, work responsibilities and standard social relationships [9], [10], [11], [12].

### Associated brain pathology and function impairments

Diffuse axonal injury - diffuse shearing of axonal pathways and small blood vessels - is one of the most common pathological feature associated with mTBI [13]. Another primary pathological feature, usually caused by a direct hit to the skull, is brain contusions, which commonly involve the frontal and anterior temporal lobes [12]. Secondary pathologies of mTBI include ischemia, mild edema, and other bio-chemical and inflammatory processes culminating in impaired regenerative/healing processes resulted from increasing tissue hypoxia [14]. Due to the diffuse nature of injury, cognitive impairments are usually the predominant symptoms, involving deficiencies in several cognitive functions, primarily memory, attention, processing speed, and executive functions, all localized in multiple brain areas. Their potent functions rely on potent network structure and connectivity between different brain areas [12], [15], [16]. We note that the diffuse nature of the mTBI injury renders the pathological damage hard to be detected by common neuroimaging methods such as CT and MRI so that diagnosis largely relies on subjective reports of the patients, as well as cognitive and quality of life tests. While diffusion tensor imaging (DTI) has the potential to detect diffuse axonal injuries, this method is still not commonly used for diagnosis of mTBI pathology.

### Rationale for hyperbaric oxygen treatment (HBOT)

The brain receives 15% of the cardiac output, consumes 20% of the total body oxygen, and utilizes 25% of the total body glucose. Still, this energy supply is only sufficient to keep about five to ten percent of the neurons active at any given time. Thus, at standard healthy condition, at any given time, the brain is utilizing almost all oxygen/energy delivered to it. The regeneration process after brain injury requires much additional energy. This is where hyperbaric oxygen treatment can help – the increased oxygen level in the blood and body tissues during treatment [17], [18], [19] can supply the energy needed for brain repair. Indeed, several previous studies have demonstrated that elevated levels of dissolved oxygen by HBOT can have several reparative effects on damaged brain tissues [3], [19], [20], [21], [22], [23], [24], [25]. Other studies revealed the beneficial effect of HBOT on the injured brain and cognitive function in animal models [26], [27], [28], [29], [30]. The elevated oxygen levels can have a significant effect on the brain metabolism, largely regulated by the glial cells (see discussion). Improved energy management leads to multifaceted repair, including activation of angiogenesis and triggering of neuroplasticity (reactivation of quiescent neurons; creation of new synapses and new axonal connections), and might even induce differentiation of neuronal stem cells [22]. The idea that HBOT can promote brain repair is reasonable and has gained experimental support, yet is still largely dismissed by the medical community as is discussed next.

### The medical community reservations

A study of the effect of hyperbaric oxygen treatment of severe brain injured patients has been published already two decades ago. Several prospective clinical trials on treatment of mTBI have been published in the last decade [31], [32], [33], and three studies published in the last two years addressed the effect of HBOT on chronic mild TBI patients [34], [35], [36]. However, the reported beneficial effects of the hyperbaric treatment were severely questioned by the medical community and triggered high skepticisms to the extent that TBI and stroke patients in the US are rarely treated by hyperbaric oxygen. The HBOT option has been dismissed by the medical community on the grounds of: 1. Lack of knowledge about the connection between metabolism and neuroplasticity. 2. Lack of randomized clinical trial with standard placebo control. 3. Sham control with room air at 1.3Atm yielded significant improvements. These issues are clarified and elaborated on in the discussion section.

### The placebo dilemma

People can sense a pressure increase beyond 1.3Atm, hence standard placebo, with normal air pressure, for HBOT could perhaps be attained by exposing the patients to normal pressure combined with falsifying stimulation (e.g., by increasing and decreasing the pressure), which generates a fictitious pressure sensation. Since breathing normal air under hyperbaric conditions leads to elevated tissue oxygen (e.g., about 50% for 1.3Atm), standard placebo could also be attained by giving the patients compressed air with sub-normal oxygen concentration. In the discussion section we explain that the first approach can be effective only for some patients and poses logistic difficulties and the second approach involves ethical issues. In an attempt to evade the placebo dilemma, a recent study of HBOT for mTBI compared the effect of 100% oxygen at 2.4Atm with the effect of room air at 1.3Atm as sham control [36]. The study found significant improvements in both groups and with slightly higher efficacy at 1.3Atm. Based on these results, the authors resented a sweeping conclusion that their study shows that HBOT has no effect on post mTBI brain damage and the observed improvements resulted from placebo associated with spending time in the hyperbaric chamber. As is discussed in great details in the discussion section, we reason that the authors reached wrong conclusions for two main reasons. First, room air at 1.3Atm cannot serve as a proper sham-control since it is not an “ineffectual treatment” (as is required from placebo) since it leads to a significant increase in the level of tissue oxygenation which has been shown to be effective [37], [38]. Second, 100% oxygen at 2.4Atm leads to too high oxygen levels which can cause inhibitory effect or even focal toxicity.

### The crossover approach

To overcome the placebo issue, a randomized crossover approach was successfully used to test the effect of HBOT in post-stroke patients at late chronic stage [3]. The advantage of the crossover approach is the triple comparison – between treatments of two groups, between treatment and no treatment of the same group, and between treatment and no treatment in different groups. Up till now, a similar prospective, randomized, crossover trial to evaluate the brain repair effect of HBOT in mTBI patients at late chronic stage has not been done.

**The aim** of our current study was to provide firm evaluation of the HBOT effects on brain activity and cognitive impairments in mTBI patients with prolonged PCS at late chronic stage.

## Methods

The study was performed as a prospective, randomized, controlled, two-group trial. The study was conducted in the hyperbaric institute and the research unit of Assaf-Harofeh Medical Center, Israel. Enrolment of patients started at 2008 and ended at 2012. All patients signed written informed consent. The protocol was approved by Assaf-Harofeh institutional review board.

### Participants

#### Inclusion

The participants were patients of age 18 years or older, who suffered mild TBI (less than 30 minutes loss of consciousness) 1-6 years prior to their inclusion. All patients experienced post concussion syndrome (PCS) and complained of impaired cognitive functions for over a year, yet brain damage was below the detection level of MRI or CT brain imaging. Only patients who reported no change in cognitive function during one month prior to the beginning of the study were included.

#### Exclusions

Exclusions were due to chest pathology incompatible with HBOT, inner ear disease, claustrophobia and inability to sign informed consent. Smoking was not allowed during the study.

### Protocol and End Points

After signing an informed consent form, the patients were invited for baseline evaluation. Included patients were randomized into two groups (1∶1 randomization): a treated group and a crossover group. The neuropsychological functions, evaluated by Mindstreams testing battery, and brain activity as visualized by SPECT (Single photon emission computed tomography), were the primary endpoints of the study. Secondary end point included quality of life evaluation by the EQ-5D questionnaire. Evaluations were made by medical and neuropsychological practitioners who were blinded to patients' inclusion in the control-crossed or the treated groups.

**Figure 1:**
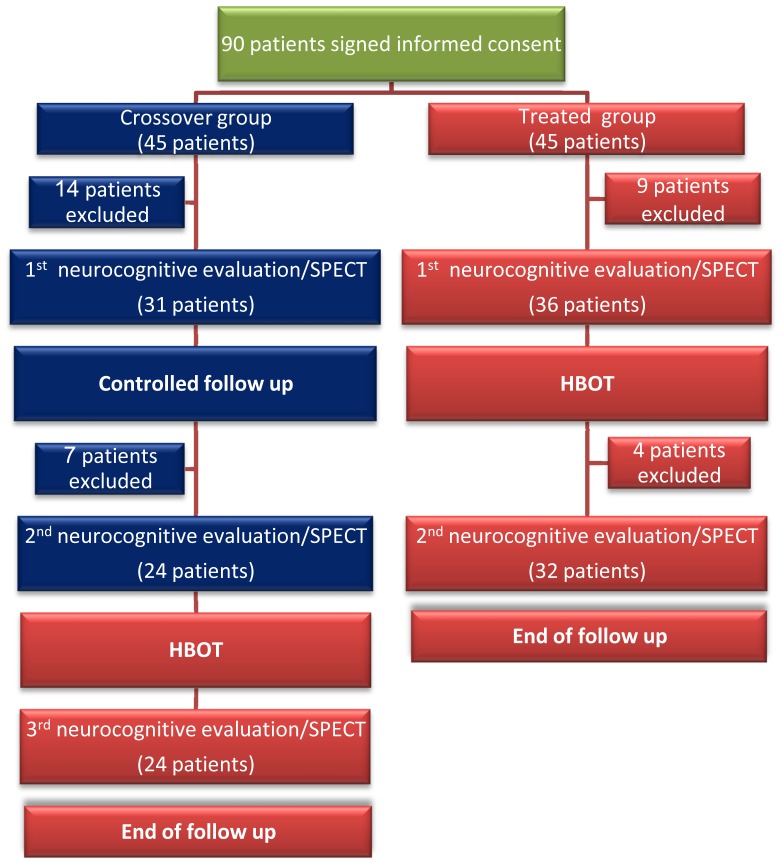
Flow chart of the patients in the study.

Patients in the treated group were evaluated twice – at baseline and after 2 months of HBOT. Patients in the crossover group were evaluated three times: baseline, after 2 months control period of no treatment, and after subsequent 2 months of HBOT (Figure 1). The post-HBOT neurological evaluations as well as the SPECT scans were performed more than 1 week (1–3 weeks) after the end of the HBOT protocol. The following HBOT protocol was practiced: 40 daily sessions, 5 days/week, 60 minutes each, 100% oxygen at 1.5ATA.

Patients were not involved in any other cognitive or rehabilitation intervention as part of the study protocol. The detailed clinical study protocol, copy of the informed consent, as well as CONSORT 2010 checklist of information are attached as supporting information (Protocol S1, Form S1, Checklist S1). We note that information regarding sample size, detectable change and power calculation parameters is included and addressed in the “statistical considerations” section in the SI1.

### Evaluation of cognitive state

#### Cognitive Indices

The state of the patients' cognitive functions was assessed in terms of the following four cognitive indices, ordered from the index associated with most fundamental (basic) functions to that associated with the higher functions: 1. ***Information Processing Speed (IPS) index***. This index is associated with the basic ability to process and respond to stimuli at different levels of speed and complexity. 2. ***Attention-related index***. This index is associated primarily with the ability to remain concentrated and respond effectively throughout relatively extended periods of time. 3. ***Memory-related index***. This index is associated with the learning of verbal and visual new stimuli, and the immediate and delayed recognition of these learned stimuli. 4. ***Executive Functions (EF) index***. This index is associated with cognitive abilities involved in the initiation, planning, organization and regulation of behavior. Each of above cognitive indices was computed as a normalized combined score of 2–3 cognitive tests from the Mindstreams Computerized Cognitive Test Battery (Mindstreams; NeuroTrax Corp., NY).

#### Cognitive tests

The Mindstreams battery includes several cognitive tests devised to check various aspects of brain capabilities. In the current study we evaluated the cognitive indices based on the scores of the 6 cognitive tests listed below, which are expected to be relevant for mild TBI. For detailed description of all cognitive tests in Mindstreams battery see [39]. The tests are:

**1.** ***Verbal memory***. Ten pairs of words are presented, followed by a recognition test in which the first word of a previously presented pair appears together with a list of four words from which the patients choose the other member of the pair. There are four immediate repetitions and one delayed repetition after 10 min.

**2.** ***Non-verbal memory***. Eight pictures of simple geometric objects are presented, followed by a recognition test in which four versions of each object are presented, each oriented in a different direction. There are four immediate repetitions and one delayed repetition after 10 min.

**3.** ***Go–No-Go test***. In this continuous performance test, a colored square (red, green, white or blue) appears randomly on the center of the screen. The patient in then asked to respond quickly only for red squares by pressing the mouse button, and inhibit his reaction to any other colored square.

**4.** ***Stroop test***. Timed test of response inhibition modified from the Stroop paper-based test. In the first phase, patients choose a colored square matching the color of a general word (for example, the word “Cat” appears in red letters, the patient must choose the red square out of two colored squares in the following screen). In the next phase (termed the Choice Reaction Time test), the task is to choose the colored square matching the name of the color presented in white letter–color. In the final (Stroop interference) phase, patients are asked to choose the colored square matching the color and not the meaning of a former color-naming word, presented in an incongruent color (for example, the word “RED” appears in green letters, the patient is asked to choose the color green and not red, a task requiring the ability to inhibit an automatic response to the meaning of the word).

**5.** ***Staged information processing test***. Timed test requiring a reaction based on solving simple arithmetic problems (pressing right/left mouse button if the answer higher/lower than 4, respectively) with three levels of information processing load (single digit, two digits addition/subtraction and three digits addition/subtraction problems), each containing three speed levels (3, 2, and 1 second for the presentation of the stimuli).

**6.** ***Catch game***. A test of motor planning that requires participants to catch a falling object on a computer screen by moving a paddle horizontally so that it can “catch” the falling object.

To assign scores, Mindstreams data was uploaded to the NeuroTrax central server. Outcome parameters were calculated using custom software blind to diagnosis or testing site. To minimize differences related to age and education, each outcome parameter was normalized and fit to an IQ-like scale (mean = 100, STD = 15) according to patient's age and education. We note that the score evaluation was based on normative data from cognitively healthy individuals collected in controlled research studies that were part of more than 10 clinical sites [40].

#### The cognitive indices’ scores

The computation of the cognitive indices based on the scores of the cognitive tests was done as follows: 1. Information Processing Speed index was computed as the combined score for the low and medium-load stages of the staged information processing test. 2. Attention index was calculated as the mean score of reaction time for Go–No-Go test and choice reaction time of the Stroop test (at second phase), mean STD of reaction time for Go–No-Go test, mean reaction time for a low-load stage of staged information processing test and mean accuracy for a medium-load stage of information processing test. 3. Memory index was computed as the mean score for total learning score (after four repetitions) and delayed recognition phase of verbal and non-verbal memory tests. 4. Executive Functions index was computed based on the scores of the Stroop test and the Go–No-Go test and the mean weighted accuracy for catch game. For more information regarding the validity of the tests and the construction of the cognitive indices, see [41], [42]. 5. In addition, we defined the individual's General Cognitive Score as the average of the scores of the four cognitive indices for each individual.

It is important to note that the above cognitive index scores were specifically designed to represent known impaired cognitive domains in mild TBI. In addition, the fact that each index is referred to more than one test-score ensures the index to be associated more with a cognitive domain score and less with a test-dependent score. We also utilized a computerized testing battery which supports the inclusion of more accurate measures such as reaction time and elimination of the bias effect of tests' administration and hand scoring. An important aspect of the tests is the inclusion of the cognitive domain of information processing speed, known to be impaired in mild TBI patients.

### Quality of life evaluation

Quality of life (QOL) was evaluated by the EQ-5D questionnaire [43]. EQ-5D essentially consists of 2 pages: the EQ-5D descriptive system and the EQ visual analogue scale (EQ-VAS). The EQ-5D descriptive system covers mobility, self-care, usual activities, pain/discomfort and anxiety/depression. The EQ-VAS records the respondent's self-rated health on a vertical, visual analogue scale [range: 0(worst)-100(best)].

### Brain Functional Imaging- SPECT

Brain single photon emission computed tomography (SPECT) was conducted with 925–1,110 MBq (25–30 mCi) of technetium-99m-methyl-cysteinate-dimmer (Tc-99m-ECD) at 40–60 min post injection using a dual detector gamma camera (ECAM or Symbia T, Siemens Medical Systems) equipped with high resolution collimators. Data was acquired in 3-degree steps and reconstructed iteratively with Chang method (μ  =  0.12/cm) attenuation correction [44].

Visual analysis was conducted by fusing pre- and post-treatment studies that were normalized to cerebellum brain activity. SPECT images were reoriented into Talairach space using NeuroGam (Segami Corporation) for identification (based on visual inspection) of Brodmann cortical areas and in order to compute the mean perfusion in each Brodmann area (BA). In addition volume rendered brain perfusion images normalized to cerebellum maximal activity were reconstructed. All SPECT analysis was done while blinded to the laboratory and clinical data. Change in perfusion in all Brodmann areas for each subject was determined by calculating the percentage difference between post-period and pre/baseline-period divided by the pre/baseline-period perfusion. An average of these perfusion changes for each Brodmann area was calculated.

### Statistical analysis

The statistical analysis was done using SPSS software (version 16.0). Continuous data is expressed as means ± standard deviations and compared by one-tailed paired t-test for intra-group comparisons and two-tailed unpaired t-test for inter-group comparisons. Effect sizes for main comparisons were calculated using Cohen's d. Categorical data is expressed in numbers and percentages and compared by χ^2^ test. P values<0.05 were considered statistically significant. All randomly allocated patients were included in the safety analysis and those with complete post-baseline assessment were included in efficacy analysis.

### Scatter plot analysis of the clinical scores

The analysis aims to better quantify and compare changes in the clinical scores, while taking into consideration the high patient-to-patient variability following Efrati et al [3]. The idea was to inspect, for each patient at each time stage, the scaled relative differences in each of the clinical scores. More specifically, we calculated for a specific patient (j) the scaled relative difference SRD_j_, defined as:
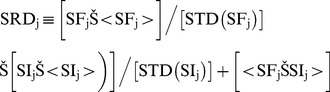
(1)


Where SF_j_ is the value of a clinical score at the end of the time stage (either treatment or control), and SI_j_ is the score at the beginning of the time stage. We note that the symbol < > indicates average over the values of the patients in the group. For example, <SF_j_> means the average of SF_j_ over all patients (j) that belong to the group. The abbreviation STD means the standard deviation between the values of the patients in the group. This analysis enables quantitative inspection of the changes in the clinical scores as is further explained in [3].

## Results

### Participants Profiles

The study included 90 screened patients, aged 18 years or older, who signed an informed consent.

#### Pre-study exclusions

Nineteen patients had their consent withdrawn before the beginning of the control/treatment period (13 in the crossover group, 6 in the treated group).

#### In-study exclusions

Four patients decided to drop out during the treatment protocol, 3 due to personal reasons and 1 due to ear problem (1 in crossover group, 3 in treatment group). Seven patients (5 in crossover group, 2 in treatment group) were excluded due to technical performance problems in their cognitive tests and 4 patients due to inconsistent use of medications (such us methylphenidate) during the tests period (2 in crossover group, 2 in treated group).

Accordingly, 56 patients (32 in the treated group and 24 in crossover group) were included in the final analysis (Figure 1). Thirty two (57%) patients were females, the mean age was 44 years (range of 21–66 years) and the time elapsed since the acute traumatic event ranged from 1–6 years with 33 months average.

The most frequent etiology of the TBI was a vehicle accident (n = 38), with some other less common etiologies (falls = 7, object hit = 6, pedestrian accident = 3, assault = 2). Baseline patients' characteristics are summarized in Table 1. As seen from this table, there was no significant difference in the included measures between the groups except for years of education, where there was a minor advantage for the treated group.

**Table 1:** Baseline patients' characteristics.

	Treated group (n = 32)	Crossover group (n = 24)	Comparison
Age (years)	42.5±12.6	45.7±10.9	p = 0.32
Gender – male	11 (34%)	13 (54%)	p = 0.07
Years of education	16.2±3.9	14.0±3.1	p<0.05
Time since injury (months)	34.6±16.7	31.7±16.3	p = 0.51
**Loss of consciousness**			p = 0.18
None	24 (75%)	14 (58%)	
<30 minutes	8 (25%)	10 (42%)	
**Etiology**			
Vehicle accident	20 (63%)	18 (75%)	
Fall	5 (16%)	2 (8%)	
Object hit	4 (12%)	2 (8%)	
Pedestrian accident	2 (6%)	1 (4%)	
Assault	1 (3%)	1 (4%)	
**Background disease**			
Hypertension (HTN)	5 (15%)	4 (16%)	
Diabetes Mellitus (DM)	2 (6%)	2 (8%)	
Hyperlipidemia	4 (12%)	3 (12%)	
Ischemic Heart Disease	0	1 (4%)	
Epileptic seizure	0	0	
Smoking	1 (3%)	0	
**Medications**			
Aspirin	2 (6%)	3 (12%)	
Glucose lowering drugs	2 (6%)	1 (4%)	
Anti-HTN	4 (12%)	3 (12%)	
Statins	3 (9%)	3 (12%)	
Anti-depressant	7 (22%)	4 (16%)	

### The Effect on Cognitive Functions

#### Changes in cognitive indices

**Figure 2:**
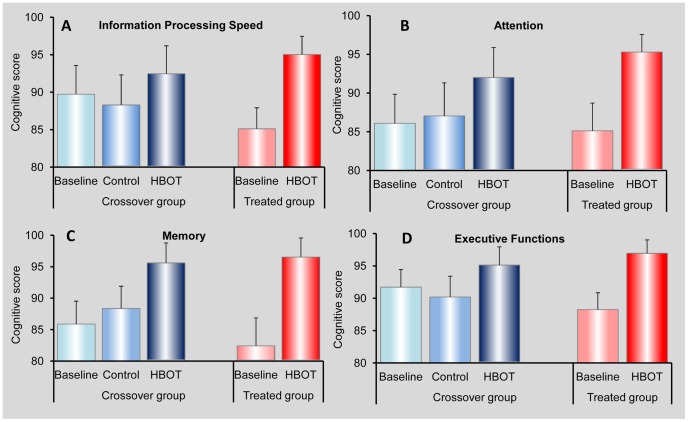
Assessment of the cognitive indices. Each patient in each group was assigned a score at baseline, after the control period (for patients in the crossover group) and after HBOT. The figures show the mean scores and standard errors for the two groups at each stage for the four cognitive indices - Information Processing Speed (A), Attention (B), Memory (C) and Executive Functions (D), as defined in the method section.

The effect of the hyperbaric oxygen treatment on the patients' cognitive functions, as assessed by the four cognitive indices, is summarized in Figure 2 and Table 2. The baseline mean cognitive scores of all four indices were close in the two groups (within the standard error) but with somewhat higher values in the treated group. The HBOT treatments of both groups led to statistically significant improvements in the mean scores of all four indices.

**Table 2:** Summary of results for Mindstreams cognitive indices scores.

	Treated group (n = 32)				Crossover group (n = 24)					
	Baseline	HBOT	P1	P2	Baseline	Control -Pre HBOT	Post HBOT	P2	P3	P4
Memory	82.43±25.15	96.54±17.18	0.567	<.0005	85.90±17.80	88.36±17.34	95.61±15.54	0.233	<0.005	0.835
Executive function	88.26±14.74	96.96±11.69	0.367	<0.0005	91.73±13.26	90.20±15.77	95.13±13.84	0.295	<0.05	0.595
Attention	85.13±20.28	95.30±12.90	0.854	<0.005	86.10±18.42	87.05±20.98	92.02±18.95	0.368	<0.05	0.443
Information processing speed	85.12±15.88	95.04±13.75	0.324	<0.0001	89.74±18.81	88.30±19.68	92.47±18.25	0.298	<0.05	0.55

Abbreviations:

Values are presented as mean ± STD. P1 stands for the p values for baseline comparison of treated and crossover group; P2 stands for the p values for comparison of the second measurement to baseline in the same group; P3 stands for the p values for comparison of pre- and post-HBOT in the crossover group; P4 stands for the p values for endpoint scores comparison following treatment in both groups. The baseline mean cognitive scores of all four indices were close in the two groups, with no significant difference. The HBOT treatments of both groups led to statistically significant improvements in the mean scores of all four indices as oppose to no significant improvement after control period alone. The tables are discussed in details in the results section.

As is apparent in Figure 2 and detailed in Table 2, a significant improvement was observed in the treated group after HBOT in all cognitive measures: Information Processing Speed (t_(31)_ = 4.20, p<0.0001), Attention (t_(31)_ = 3.26, p<0.005), Memory (t_(31)_ = 4.13, p<0.0005) and Executive Functions (t_(31)_ = 3.72, p<0.0005). Effect sizes were medium to large: the Cohen's d measures [45] were 0.74, 0.57, 0.73 and 0.66, respectively.

No significant improvement was noticed in the crossover group during the control period: Information Processing Speed (t_(23)_ = 0.53, p = 0.298), Attention (t_(23)_ = 0.33, p = 0.368), Memory (t_(23)_ = 0.74, p = 0.233) and Executive Functions (t_(23)_ = 0.54, p = 0.295). However, a significant improvement following HBOT was noticed in the crossover group as well: Information Processing Speed (t_(23)_ = 1.98, p<0.05), Attention (t_(23)_ = 2.29, p<0.05), Memory (t_(23)_ = 3.21, p<0.005) and Executive Functions (t_(23)_ = 2.26, p<0.05). Effect sizes were medium to large, with Cohen's d measures of 0.40, 0.47, 0.65 and 0.46, respectively. Note that t_(31)_ and t_(23)_ correspond to N-1, where N = 32 and N = 24 are the number of patients in the treated and crossover group, respectively.

#### Assessment of a general cognitive score

**Figure 3:**
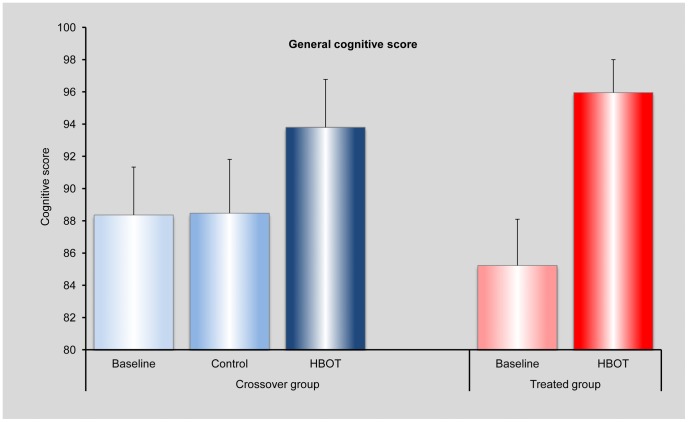
Assessments of the general cognitive score. The figure shows the level of the general cognitive score (defined in the text) for the crossover group at baseline, after the control period and after HBOT, and for the treated group at baseline and after HBOT.

The changes in the four cognitive indices presented in Figure 2 and in Table 2 show noticeable variability. For example, the mean values of the Information Processing Speed and Executive Functions indices decreased during the control period of the crossover group, while the corresponding mean values of the Attention and Memory indices increased. Figure 3 shows the mean values of the individual general cognitive scores, with standard error, for the treated and crossover groups at each evaluation stage: baseline and post-HBOT for both groups, and after the control for the crossover group. It can be seen that the cross group had the same general score at baseline and after the control period. This value seems higher than the score of the treated group at baseline – ∼88 vs. 85, and the post-HBOT general cognitive score of the treated group seems higher than that of the crossover group – ∼96 vs. 94. While these differences are within the standard error, they still give rise to what appears to be significant differences in the level of changes (post- vs. pre-HBOT) between the crossover and the treated groups: 6 points for the crossover group vs. 11 for the treated group.

#### Examining the relative changes

**Figure 4:**
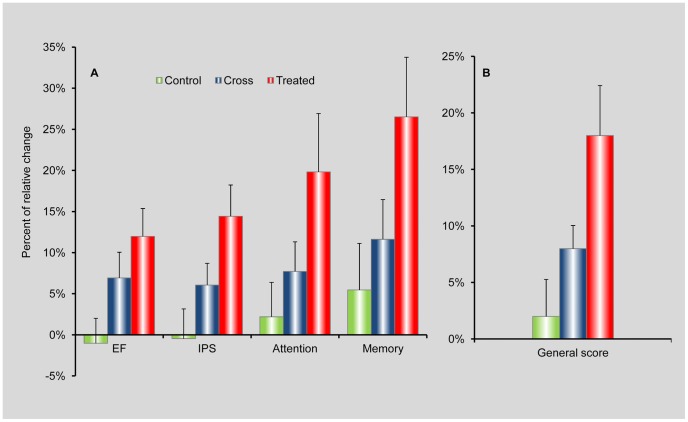
Assessment of the relative changes. A) The relative changes (as defined in the text) for the four cognitive indices. The changes are shown for the crossover group following the control period (green bars) and HBOT (blue bars), and for the treated group following HBOT (red bars). B) Relative changes in the general cognitive score for the same three cases as in (A).

There is a high patient-to-patient variability in the cognitive indices, with scores ranging from 20 to 120. The magnitude of the change in a cognitive score has different implications for patients at low or high base levels. Hence, we inspect the effect of the HBOT on the relative changes, i.e. the change relative to the base value. We calculated, for each person, the relative change in each of the cognitive indices for each period (control and HBOT for the crossover group and HBOT for the treated group). In Figure 4A we show the mean relative changes in all four cognitive indices for the crossover group following the control period and following HBOT, and for the treated group following HBOT. In Figure 4B we show the mean relative changes in the general cognitive score for the same three periods. We note that calculating the mean of the relative changes is more informative than calculating the changes in the mean values, especially for small groups with high patient-to-patient variability.

Looking at the relative changes elucidates the improvements after the HBOT period vs. the control period of the crossover group. However, it also amplifies the differences mentioned earlier between the crossover and treated groups: the bigger relative changes in the treated group vs. the crossover group reflect the fact that the baseline values of the treated group were lower and the post-HBOT values were higher in comparison to the corresponding values of the crossover group.

#### Scatter plot analysis of the cognitive indices

**Figure 5:**
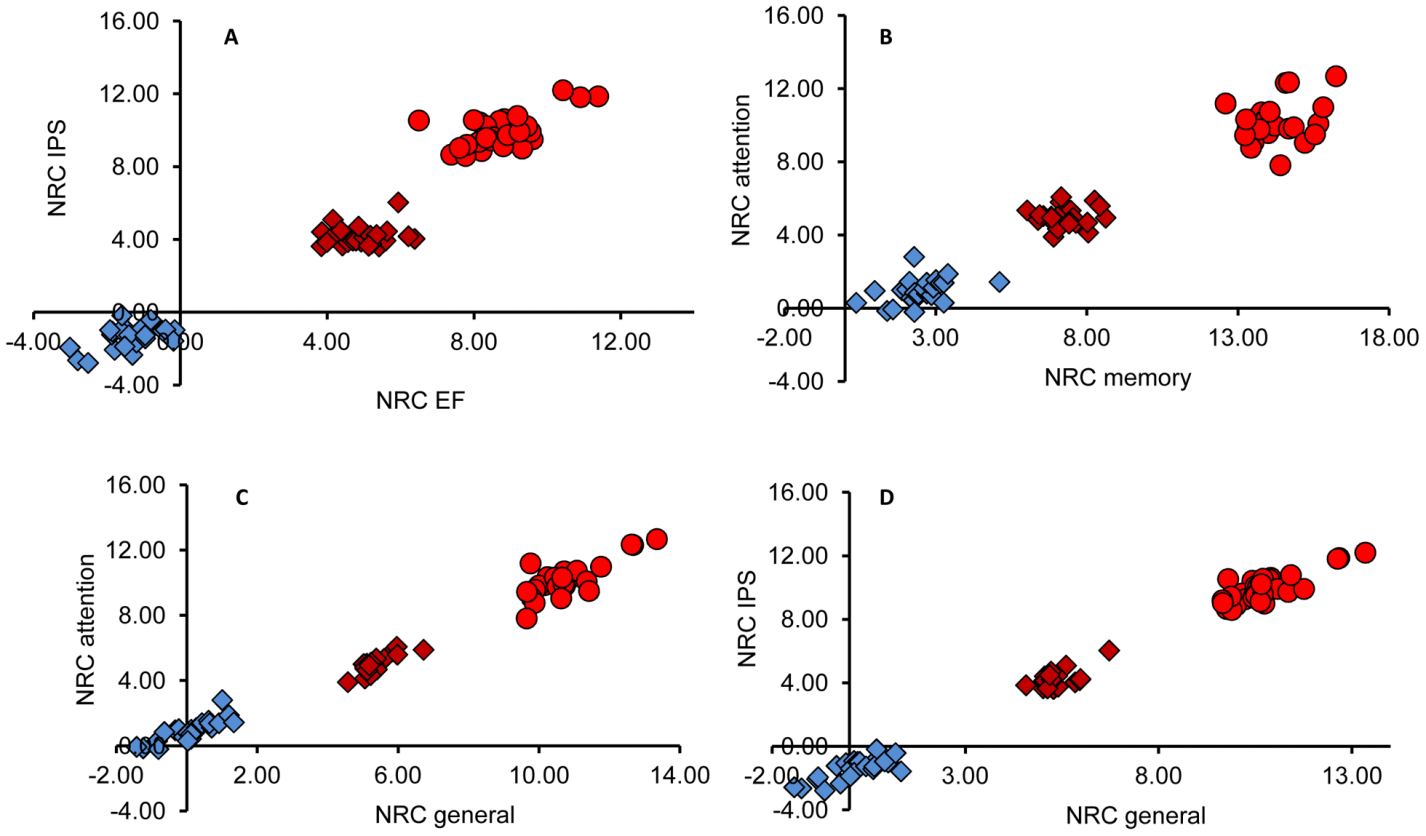
Scatter plot analysis of the changes in cognitive indices. The scatter plots show the normalized relative changes (NRC) as defined in the methods section and explained in the text above. A) Scatter plot for the changes in IPS as function of changes in EF. B) Scatter plot for changes in Attention as function of Memory. The changes in the Attention and in the IPS as function of the General cognitive score are shown in (C) and (D), respectively. Circles are for the treated group and diamonds are for the crossover group. The color code is: Red for changes during HBOT and blue for changes during control.

As mentioned in the methods section, the analysis aims to present the mean relative changes in cognitive indices while superimposing information regarding the patient-to-patient variability. For that we calculated, for each patient (i), the normalized relative change NRC(i). Next, we calculated for each group (control and HBOT in the crossover group and HBOT in the treated group) the locations of the mean departures from baseline. Finally, we marked the location of each patient (i) at a distance NRC(i) from the location of his/her group's mean difference (see methods section for details). Typical results are shown in Figure 5. More specifically, we show the scatter plots for Information Processing Speed vs. Executive Functions (Figure 5A), for Attention vs. Memory (Figure 5B), for Attention vs. General cognitive score (Figure 5C), and for IPS vs. General cognitive score (Figure 5D). The results illustrate the differences between the three cases (control and HBOT for the crossover group, and HBOT for the treated group) which form three distinct clusters. Also clear in all the figures is the linear dependence between the changes in the different cognitive indices (similar results are also obtained for the other combinations, e.g. Memory-IPS, Attention-IPS, Memory-EF and Attention-EF). Interestingly, the scattering of the individual patients also follows the linear line for the scatter plots of the specific cognitive indices as function of the general cognitive score (Figures 5C and 5D). These linear dependences demonstrate high consistency between the changes of the different cognitive indices for each patient. As such, the analysis provides valuable test for the validity of the test performances and the validity of the general cognitive score.

### The Effect on quality of life

The effect on the QOL is summarized in Table 3. The EQ-5D score significantly improved following HBOT in the treated group (t_(31)_ = 7.41, p<0.0001) and in the crossover group after HBOT (t_(23)_ = 6.17, p<0.0001). As expected, there was no improvement in the EQ-5D score in the crossover group following the control period. During the control period, we have noticed some reduction in this group with respect to the patients' subjective perception of their quality of life (t_(23)_ = 2.60, p<0.01). Similar results were obtained for the EQ-VAS evaluations as summarized in Table 3. More specifically, the EQ-VAS score significantly improved following HBOT, both in the treated group (t_(31)_ = 4.86, p<0.0001) and in the crossover group following treatment (t_(23)_ = 4.79, p<0.0001), while there was no significant improvement following the control period (t_(23)_ = 0.32, p = 0.373). Details are presented in Table 3.

**Table 3:** Summary of results of quality of life questionnaire (EQ-5D and EQ-VAS).

	Treated group (n = 32)				Crossover group (n = 24)					
	Baseline	HBOT	P1	P2	Baseline	Control-Pre HBOT	Post HBOT	P2	P3	P4
EQ-5D	7.87±1.36	6.48±1.07	0.615	<0.0001	7.70±1.11	8.06±1.05	6.75±1.06	<0.01	<0.0001	0.362
EQ- VAS	5.03±2.31	6.62±2.45	0.696	<0.0001	5.26±1.70	5.21±1.66	6.39±1.80	0.373	<0.0001	0.696

Abbreviations:

Values are presented as mean ± STD. P1 stands for the p values for baseline comparison of treated and crossover group; P2 stands for the p values for comparison of the second measurement to baseline in the same group; P3 stands for the p values for comparison of pre- and post-HBOT in the crossover group; P4 stands for the p values for endpoint scores comparison following treatment in both groups. EQ-5D as well as the EQ-VAS scores significantly improved following HBOT, both in the treated group and in the crossover group following treatment, while there was no significant improvement following the control period.

#### Examining the relative changes

**Figure 6:**
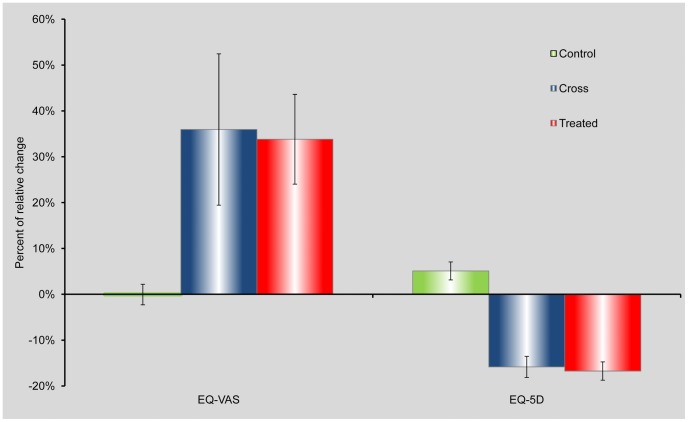
Assessments of the mean relative changes and standard errors in quality of life measurements. The changes are shown for the crossover group following control period (green bars) and following HBOT (blue bars), and for the treated group following HBOT (red bars). Note that, according to the questionnaire structure, in the EQ-5D measurement improvement is reflected as score decrease, hence the negative values of change.

Figure 6 presents the mean relative changes and standard errors in both measurements of quality of life for the treated group following HBOT and for the crossover group after the control period and after HBOT.

### SPECT assessments of brain activity

#### Motivation

Since mTBI involves a diffuse structural and/or physiologic/metabolic derangement [46], [47], [48], patients with mTBI have more frequent and more extensive areas of brain damage than can be seen by anatomical imaging (conventional CT and MRI scans). The preferred brain imaging methods are thus functional/metabolic (SPECT, PET, CT perfusion, and functional MRI). In order to achieve greater validity of the results, cognitive function and SPECT analysis were done by a blinded evaluation and evaluator: the cognitive function tests were done by a computerized validated method and the SPECT analysis was blind to patients' participation in treated/crossover group.

**Figure 7:**
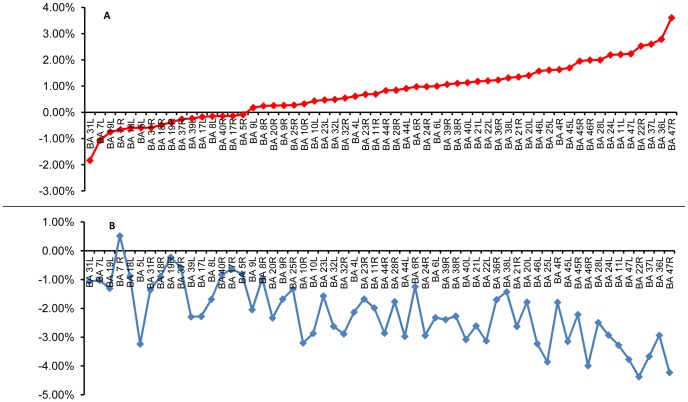
Distribution of the Brodmann areas relative SPECT CBF changes. The change for each BA represents and averaging of the relative changes of all the patients as explained in the text. The results show a clear difference between the control and the HBOT periods. We note that the higher variations for the control period are associated with the fact that the averaging in this case is over 24 patients (the crossover group), while for the HBOT period the averaging is over all 55 patients.

Brain SPECT evaluations were completed for 31 patients in the treated group (one patient from the treated group did not complete two SPECT scans) and for 24 in the crossover group. In supporting information (Table S1) we present detailed statistics of the SPECT results for all Brodmann areas (BA) of all the tested patients. The results revealed a significant increase in brain activity (perfusion) following the hyperbaric oxygen treatments in both groups compared to the change during the control period of the crossover group. A summary of the results is presented in Figure 7. To construct the figure we calculated, for each patient, the relative change in the SPECT measured brain activity during each phase of the trial. The relative change was defined as the difference in normalized activity (relative to the cerebellum activity, see SI4) between the end point and the start point divided by the activity at the start. We calculated, for each BA, the average changes over all patients that underwent HBOT (both treated and cross groups). These results correspond to the red curve in Figure 7. The blue curve represents the results of similar calculations for the control period (averaging for each BA over all the results of all patients in the crossover group during the control period).

The results revealed significant improvement in brain perfusion following HBOT in the frontal and temporal areas, including: inferior frontal gyrus (BA 45), the middle frontal area (BA 46), parts of the orbito-frontal cortex (BA 47 and 11), most of the temporal cortex (BA 36, 37, 38, 20, 21, 22, 28), and parts of the Cingulate gyrus (BA 24, 25). These fronto-temporal regions, responsible for the cognitive functions, are the most affected in head trauma [12]. The temporal lobe plays a significant role in memory and learning skills [49], [50], [51]; the frontal lobe is associated with attention and executive functions [52], [53], and the cingulate gyrus plays an important role in emotions and cognitive self regulation [54], [55], [56]. The SPECT results of elevated activity in these Brodmann areas are consistent with the improvement in cognitive indices following HBOT. Further below we show specific examples of SPECT results for four patients (two from the treated group and two from the crossover group).

### Representative examples

#### Example 1

**Figure 8:**
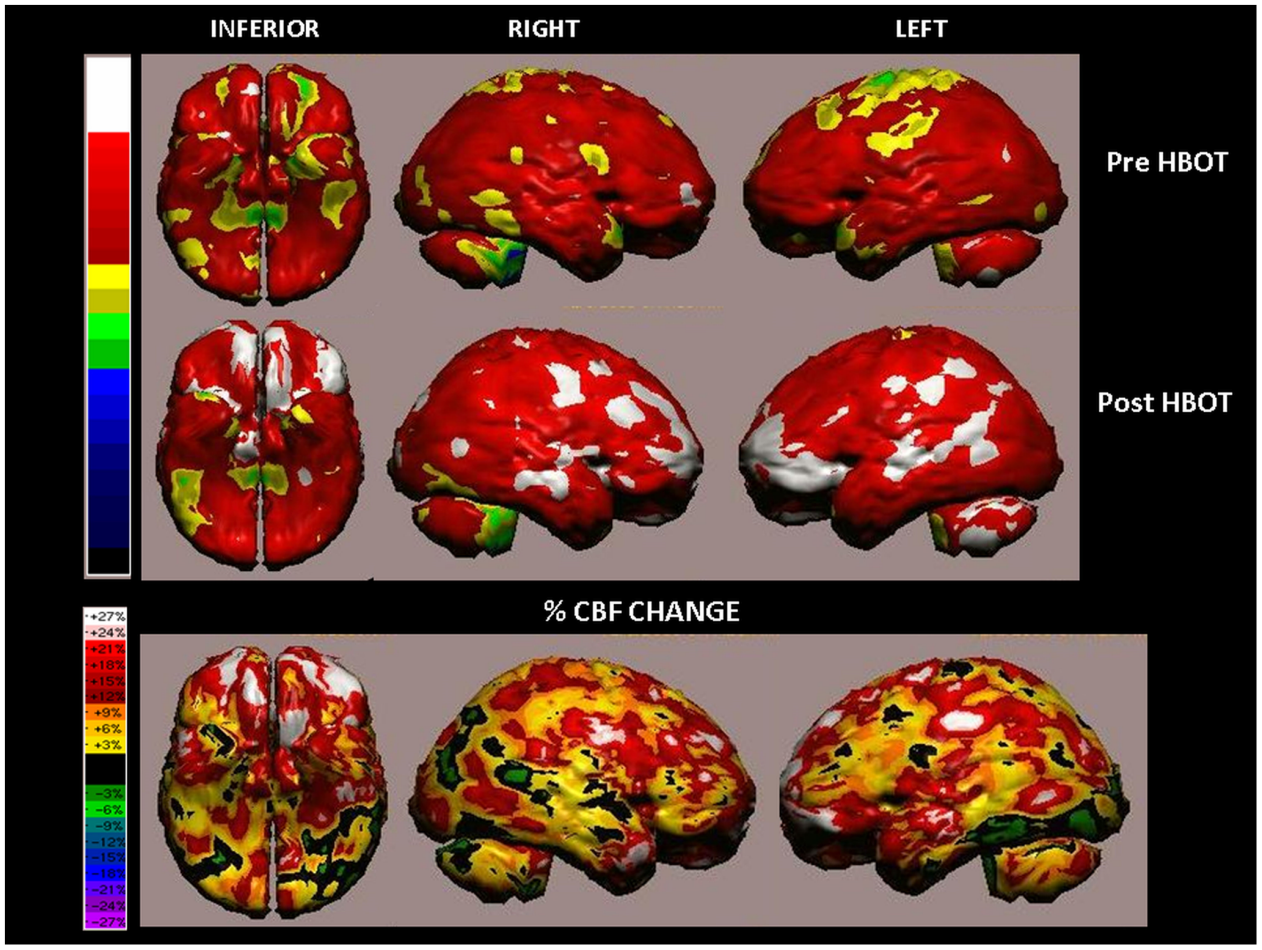
Volume rendered Brain SPECT perfusion maps of Example 1, a 51-year-old woman from the treated group suffering mTBI that had occurred 2 years prior to inclusion in the study. Comparison of the baseline activity (upper row) with the post HBOT activity (middle row) and the CBF changes (bottom row) demonstrated significant improvements after HBOT in bilateral orbito-frontal and lateral-parietal regions and left ventro-lateral-frontal region correlating to BAs 45, 47, and 11.

SPECT analysis of a 51-year-old woman from the treated group (Figure 8). The patient suffered from cognitive impairments due to mTBI as a result of a fall in a moving bus 2 years prior to inclusion in the study. The patient experienced no loss of consciousness and CT imaging did not reveal anatomic damage. The patient's main complaints included headaches, dizziness, memory and concentration problems, and random mood swings. The patient was a manager in a private business, and since the injury was having difficulties following and completing daily activities and routine. Following HBOT, the patient reported significant improvement in every aspect of daily functioning. The patient's cognitive indices demonstrated significant improvements following treatment: increase of 3.5 STD in Memory (56 pre-HBOT to 108 post-HBOT), 2 STD increase in Attention (47 to 81), 1.5 STD increase in Executive Functions (65 to 85) and a 0.7 STD increase in Information Processing Speed (85 to 95).

#### Example 2

**Figure 9:**
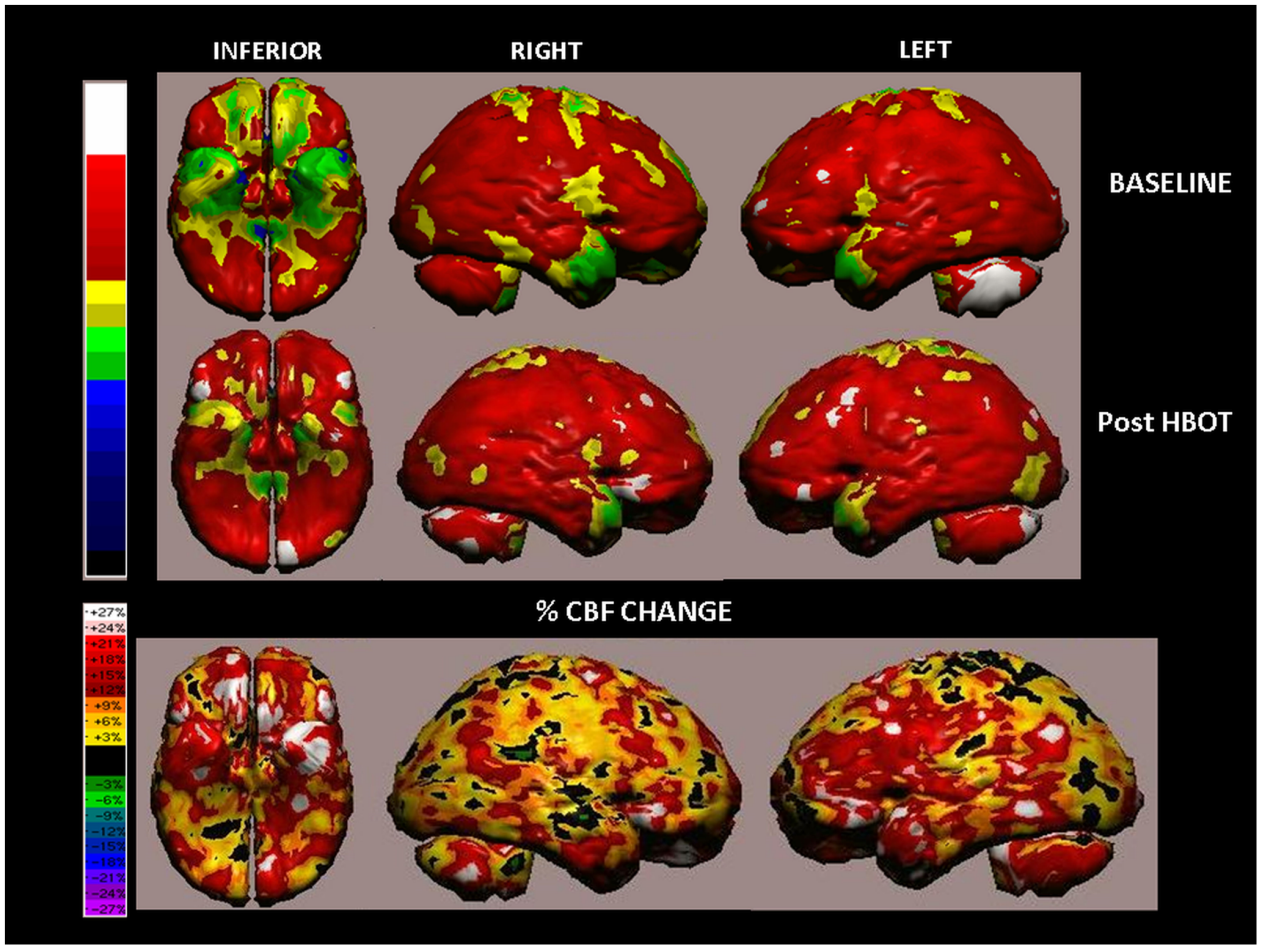
Volume rendered Brain SPECT perfusion maps of Example 2. The results are of a patient in the treated group, suffering mTBI that had occurred 1 year prior to inclusion in the study. Comparison of the baseline activity (upper row) with the post-HBOT activity (middle row) and the CBF changes (bottom row) demonstrated significant improvements after HBOT in bilateral orbito-frontal regions, the medial aspect of the temporal lobes and the temporal poles that correspond to BAs 11, 25, 27, 28 and 38.

SPECT analysis of a 46-year-old woman from the treated group, following mTBI as a result of a car accident 1 year prior to inclusion in the study. The patient's main complaints were related to her memory and concentration capabilities, as well as dizziness and tinnitus that interfered with her daily functioning. Following HBOT, the dizziness and tinnitus disappeared, in addition to significant improvement in all cognitive domains. The patient's cognitive indices demonstrated significant improvements following treatment: increase of 1.5 STD in Memory (57 pre-HBOT to 78 post-HBOT), 1 STD increase in Attention (88 to 104), 1.5 STD increase in Executive Functions (82 to 102) and 0.6 STD increase in Information Processing Speed (85 to 95). SPECT images of the patient at baseline and following HBOT are shown in Figure 9. The percentage of CBF change demonstrated significant improvements after HBOT in parts of the frontal and temporal lobes, in agreement with the improvements in neurological functions.

#### Example 3

**Figure 10:**
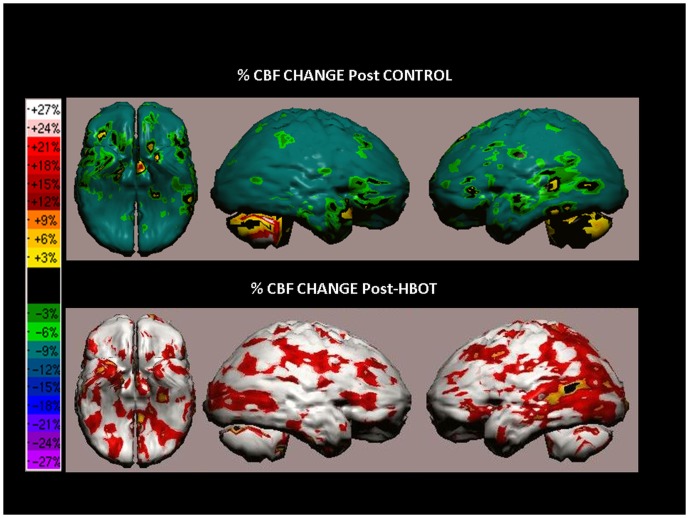
Volume rendered Brain SPECT images representing the percentage of CBF change post control period and post HBOT of the cross group patient described in example 3. As can be clearly seen, the improvement in perfusion following HBOT was significantly high in most areas of the brain as opposed to insignificant change following the control period. The most significant improvements were in both frontal and temporal lobes and right parietal lobe.

SPECT analysis of a 44-year-old man from the cross group, suffering from cognitive impairments due to mild TBI as a result of a fall 2 years prior to treatments. The patient complained mainly on short and long term memory difficulties, attention and concentration problems, decline in naming abilities, as well as headaches, dizziness, anxiety and sleep problems. Following HBOT, the patient experienced significant improvements in most aspects, including concentration and memory, headaches, dizziness, mood and sleep. The patient's cognitive indices demonstrated significant improvements in Executive Functions after treatment: baseline 60, after control 63 and post-HBOT 74. SPECT images of the patient after the control period and following HBOT are shown in Figure 10. The CBF change was not significant after the control period but substantial improvement after HBOT in most of bilateral frontal and temporal lobes and right parietal lobe.

#### Example 4

**Figure 11:**
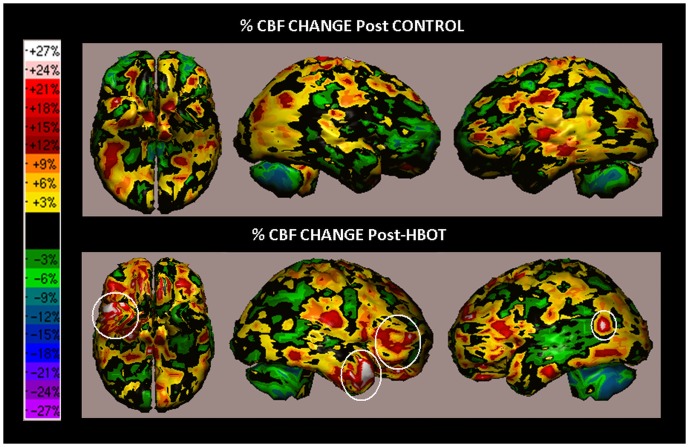
Volume rendered Brain SPECT images representing the CBF change (in percentage) post control period and post HBOT of the cross group patient described in example 4. The overall changes after the control period and the HBOT show normal variations of brain perfusion in the -10% to +10% range (from green to orange colors). However, close inspection reveals localized significant changes (white circles) in the in the right temporal pole and in the right dorso-lateral area. These changes in perfusion are in good agreement with the improvements in the cognitive indices as the SPECT detected changes correspond to Brodmann areas 45–46, 11, 38 and 39.

SPECT analysis of a 39-year-old woman from the cross group, suffering from cognitive impairments due to mild TBI as a result of a car hit as a pedestrian, 2 years prior to treatments. The patient's complaints included dizziness, nausea, fatigue and decrease in memory and concentration abilities. The patient also had troubles in performing every-day activities, such as attaining the grocery store, or reading the newspaper. The patient was working as a nurse and could not continue to perform her work after the accident, due to the impaired abilities. The patient's cognitive indices demonstrated significant improvements following treatment in comparison to the changes during the control period: Executive Functions at baseline, after control and post HBOT were 77, 71 and 88 respectively, and Attention scores were 62, 64 and 78 respectively. SPECT images of the patient, after the control period and following HBOT are shown in Figure 11. At first global look, the CBF changes after HBOT seem to be similar to the changes after the control period demonstrating no significant change. However, a closer inspection of the SPECT images reveal local improved perfusion in the right anterior temporal and right dorso-lateral frontal areas. This example is shown to demonstrate that even for patients with relatively mild cognitive improvements there is good correspondence between the change in the cognitive tests and the SPECT results.

## Discussion

We presented a prospective, randomized and controlled cross over study of the effect of HBOT with 100% oxygen at 1.5Atm on mTBI patients at late chronic stage. The results clearly demonstrate that HBOT can induce neuroplasticity and significant brain function improvement in mild TBI patients with prolonged Post-Concussion-Syndrome at late chronic stage, years after brain injury. Additional statistical validation using simulated randomizations is available as supporting information (Text S1).

### Linking elevated oxygen, metabolism and brain activity to neuroplasticity

The changes in SPECT images after treatment indicate that HBOT led to reactivation of neuronal activity in stunned areas that seemed normal under CT and MRI imaging. While SPECT imaging has a limited spatial resolution (compared, for example, to fMRI), the changes in activity were sufficiently robust to be clearly detected by the SPECT images.

Recently, Kan et al. [57] discussed the need for potent interventions, such as elevated tissue oxygen, capable of repairing microenvironment alterations after mTBI (e.g impairments in vascular changes, in cerebral blood flow and in perfusion), leading to reduced oxygen availability followed by reduced metabolism, which in turn leads to reduced neuronal activity, loss of synapses and tampered neuronal connectivity.

The observed reactivation of neuronal activity in the stunned areas found here, along with similar results in post-stroke patients [3], imply that increasing the plasma oxygen concentration with hyperbaric oxygenation is a potent means of delivering to the brain sufficient oxygen for tissue repair. HBOT might initiate a cellular and vascular repair mechanism and improve cerebral vascular flow [34], [58], [59], [60]. More specifically, HBOT induces regeneration of axonal white matter [61], [62], [63], [64], has positive effect upon the myelinization and maturation of injured neural fibers [65], and can stimulate axonal growth and increase the ability of neurons to function and communicate with each other [66]. In addition, HBOT was found to have a role in initiation and/or facilitation of angiogenesis and cell proliferation processes needed for axonal regeneration [67].

At the cellular level, HBOT can improve cellular metabolism, reduce apoptosis, alleviate oxidative stress and increase levels of neurotrophins and nitric oxide through enhancement of mitochondrial function (in both neurons and glial cells). Moreover, the effects of HBOT on neurons can be mediated indirectly by glial cells, including astrocytes [23]. HBOT may promote the neurogenesis of endogenous neural stem cells [24]. With regard to secondary injury mechanisms in mTBI, HBOT can initiate vascular repair mechanism and improve cerebral vascular flow [58], [59], [68], [69], promote blood brain barrier integrity and reduce inflammatory reactions [28] as well as brain edema [20], [21], [22], [26], [34], [70]. A drawback to the above-mentioned findings is that the different effects have been tested at different experimental setups and while utilizing different protocols of HBOT. However, it is well noticed that there is at least one common denominator to all repair/regeneration mechanisms: they are all energy/oxygen dependent. It might be possible that HBOT enables the metabolic change simply by supplying the missing energy/oxygen needed for those regeneration processes.

### Rationale for testing the HBOT effect on patients at late chronic stage

As stated in the introduction, the crossover approach is adopted in order to avoid the inherent difficulties associated with randomized HBOT trial while practicing standard placebo (see Text S1). The placebo dilemma and the rationale for a crossover approach are further discussed below following the rationale to selecting patients at late chronic stage. First, as explained in [3], applying hyperbaric oxygen in the acute or early phase after brain injury makes it almost impossible to signify and assess the HBOT effects vs. the effects of the spontaneous natural repair mechanism that are effective at this stage. Moreover, in some patients the elevated oxygen can inhibit natural regeneration or even cause toxicity. In [3] it was proposed that this might explain the contradictive results in studies using HBOT at early stage after stroke [71], [72], [73], [74], [75]. One can assume that any added energy during the degenerative stage, immediately after brain injury, could further increase the unwanted, post-injury damage. On the other hand, elevated oxygen supply during the regenerative stage would supply the energy needs for the innate brain repair processes. Second, as also explained in [3], patients at the chronic late stage demonstrate neurological stability with low probability of spontaneous changes unrelated to treatment. Third, typically, patients at this stage, years after injury, have already gone through rehabilitation programs. These programs, which attempt to attend mainly to the cognitive dysfunction following the injury, are commonly based on behavioral compensation methods (such as attention training drills, teaching memory, planning strategies and usage of external aids [16], [76], and have limited patient-specific success in repair of mTBI impaired brain function [77]. Therefore, studies at the late chronic stage allow assessment of the power of the HBOT approach to achieve brain function improvements in addition to, rather than instead of, the standard rehabilitation programs.

### The placebo dilemma and debate

There are inherent ethical and logistic difficulties in handling the sham control in HBOT trial according to the standard placebo definition: “*Medically ineffectual treatment for medical conditions intended to deceive the recipient from knowing which treatment is given*”. First, the minimal pressure for the patients to sense pressure increase is 1.3Atm. Second, breathing regular air under hyperbaric conditions of 1.3Atm leads to more than 50% elevation in tissue oxygenation. There are many case reports illustrating significant effects due to small increases in air pressure, including effects on the brain [38], [78], [79], [80]. Moreover, even a slight increase in partial pressure, such as to 1.05 ATM at altitude 402 m below sea level (the Dead Sea), can lead to noticeable physiological effects [81], [82], [83], [84], [85]. Since 50% elevation in tissue oxygen can have significant physiological effects, treatment with room air at 1.3Atm is not an “ineffectual treatment” as is required from a proper sham control. Yet, a recent randomized, controlled trial on mTBI patients by Wolf et al [36], used room air at 1.3Atm as sham control for treatment with 100% oxygen at 2.4Atm. Both groups revealed significant improvements in cognitive symptoms and in the measure of post traumatic stress disorder (PTSD). We find these results very important: they actually demonstrate that the significantly less expensive and logistically simpler treatment of mTBI patients with mild HBNO2 (mild hyperbaric pressure of 1.3Atm and regular air) can lead to meaningful improvements. Our interpretation is based on previous studies demonstrating that mild HBNO2 conditions can be effectual treatment. The authors of that study presented a very different interpretation. Overlooking the fact that mild HBNO2 can be an effectual treatment, they regarded it as sham control and concluded that the observed improvements must be due to placebo, and that HBOT has no therapeutic effect on mTBI patients. In other words, they implicitly assumed that bringing the patients many times to spend long duration in the hyperbaric chamber can trigger such a powerful placebo effect that it can lead to a significant repair of chronic brain damage due to mTBI. Remembering that for mTBI patients (with intact macro vascular bed), breathing 100% oxygen at 2.4ATA generate very high oxygen levels in tissues, which can cause an inhibitory effect or even focal toxicity, it is conceivable that HBOT using 2.4 ATA can be less effective than 1.3 ATA or other lower levels of pressure [86]. Future studies are needed to test this issue by evaluating the specific dose response in post mTBI patients.

A potential way to comply with standard placebo could be to expose the patients to normal pressure combined with falsifying stimulations (e.g., by increasing and decreasing the pressure), which generates a fictitious pressure sensation. This approach poses non trivial logistic difficulties. Some patients, especially in long-term repeated treatments, can detect pressure fluctuations. Another potential way to avoid the increase in tissue oxygen at 1.3Atm in order to attain a standard placebo is to let the patients breath air with lower than normal oxygen level. Obviously, this is an unsuitable approach, as it involves ethical issues and leaves an open question with regards to the pressure effect. Nevertheless, Cifu et al [35] conducted a randomized blinded clinical study in which 2.0 ATA with 10.5% oxygen was used as the sham control. More specifically, the patients were at 2.0 ATA but were randomly assigned to one of three groups breathing either 10.5%, 75% or 100% oxygen to mimic normal air at 1.0 ATA, 100% air at 1.5 ATA and 100% air at 2.0 ATA, respectively. The authors concluded that: “This study demonstrated that HBO2 at either 1.5 or 2.0 ATA equivalent had no effect on post-concussion symptoms after mild traumatic brain injury when compared with sham compression”. Unfortunately, the HBOT effect in this study was assessed merely based on the self-administered Rivermead Post-Concussion Symptoms Questionnaire (RPQ) which is known to display several flaws in implementation and in its ability to accurately reflect test-taker experience. Moreover, interpretation and accuracy of the RPQ can vary widely due to self-administration and the various confounding variables involved [87]. Put aside this weakness, the study suffers from a logical flaw: The authors mention that their study was motivated by the results of Wolf et al. [36], and they accepted the interpretation that any observed improvements should be a reflection of placebo effect and have nothing to do with the HBOT. If indeed, placebo can be so powerful in mTBI patients, one would expect that stress related to the idea of breathing half the normal level of air may trigger powerful negative placebo effect.

### Rationale for the crossover approach

In the current study we tested the effect of 1.5 ATA using the crossover approach. As stated in the introduction, the approach is adopted in order to avoid the inherent difficulties associated with conducting HBOT trial while practicing standard placebo. The crossover approach involves two groups – a treated group in which the patients went through two months of 40 HBOT sessions, and a crossover group in which the patients first went through two month of no treatment followed by two months of HBOT sessions. The advantage of the crossover approach is the triple comparison – between treatments of two groups, between treatment and no treatment of the same group and between treatment and no treatment in different groups (see Text S1). For both groups, the HBOT sessions induced statistically significant improvement in cognitive functions (according to four cognitive indices: Information Processing Speed, Attention, Memory and Executive functions), in brain activity (according to SPECT imaging) and in quality of life (according to the EQ-5D and the EQ-VAS scores), compared to the control period of the crossover group. To gain better validity of the results, we used the scatter plot analysis of the changes of the cognitive indices in terms of the corresponding scaled relative changes. The scatter plots (figure 5) show correlations in the improvements of the different indices both for the group means and the individual patients. The good correspondence between the improvements in the cognitive indices, the quality of life scores and the elevated brain activity as revealed by the SPECT imaging, which was done in a completely blinded fashion, further substantiates the clinical findings.

## Implications

Combined with previous studies of the HBOT effects on TBI and CVA patients, the results presented here show that treatment with hyperbaric oxygen can significantly repair the chronically impaired brain functions and dramatically improve the quality of life of these patients. Yet, HBOT did not become a common acceptable treatment for TBI and CVA, largely because of the debate regarding the placebo issue and the optimal time for administration. Additional larger scale clinical studies are required to asses if and to what extent placebo effects might be operative. However, since the improvements are significant with no significant side effects, it seems reasonable to let patients benefit from HBOT now rather than wait until future studies are completed.

We foresee that the future oxygen-pressure dose-response studies, described in the discussion section, will have significant therapeutic implications. In particular, we expect that HBOT treatment with room air at 1.3ATA will have significant brain repair effects, and its effect should be compared with the 1.5ATA protocol used in this study.

In the current study, the HBOT effects were assessed shortly after treatment ended. Future follow-up studies are needed in order to investigate the durability of the effect. It might be that some patients will need more than 40 HBOT sessions. The issue of how to optimize patient-specific protocol is important subject for future research.

**In conclusion**, this study provides, for the first time, convincing results based on a crossover study, demonstrating that HBOT can induce neuroplasticity and significant brain function improvements in mild TBI patients with prolonged Post-Concussion-Syndrome at late chronic stage, years after injury. The results call for better understanding of how to set the optimal HBOT protocol for the specific patients and how to determine which patients benefit the most from this treatment. The findings reported here bear the promises that HBOT can be effective in treating other brain impairments, like easing PTSD symptoms or repairing radiation damage. It is also reasonable to expect that HBOT can help slow down or even reverse metabolic disorders associated neurodegenerative diseases.

## Supporting Information

Table S1 **SPECT based measurements of changes in brain activity.** This SI includes data regarding the SPECT imaging for all the patients (Table S1.1–S1.3). The data was normalized according to Cerebellum activity, and the relative change percentage from baseline was calculated for each subject for each Brodmann area. Average and STD of all subjects were then calculated for each BA. The data is available for all three groups of subjects - control group after waiting period, control group after HBOT (crossover), and treated group after HBOT. Doing so ease associating the changes in SPECT measurements of brain activity with the assessed changes in the cognitive indices.
(PDF)

Protocol S1 **Clinical study protocol.**
(DOCX)

Form S1 **Informed consent form (English translation).**
(DOCX)

Checklist S1 **CONSORT 2010 checklist.**
(DOCX)

Text S1 **Crossover approach and simulated randomization.**
(DOC)
