# Supplementary material for: Hyperbaric Oxygen Therapy Can Improve Post Concussion Syndrome Years after Mild Traumatic Brain Injury - Randomized Prospective Trial
Source: PLoS One. 2013 Nov 15;8(11):e79995. doi: 10.1371/journal.pone.0079995 (PMC3829860; doi:10.1371/journal.pone.0079995)
Supplement: Table S1 — SPECT based measurements of changes in brain activity. This SI includes data regarding the SPECT imaging for all the patients (Table S1.1–S1.3). The data was normalized according to Cerebellum activity, and the relative change percentage from baseline was calculated for each subject for each Brodmann area. Average and STD of all subjects were then calculated for each BA. The data is available for all three groups of subjects - control group after waiting period, control group after HBOT (crossover), and treated group after HBOT. Doing so ease associating the changes in SPECT measurements of brain activity with the assessed changes in the cognitive indices. (PDF) [file pone.0079995.s001.pdf]

Table S1-1. The relative change percentage of SPECT imaging data for patients in the control group after control period, in each Brodmann area.

	subject #																									
	1	2	3	4	5	6	7	8	9	10	11	12	13	14	15	16	17	18	19	20	21	22	23	24	**AVG**	**SD**
BRODMANN AREA 4 LEFT	-10.02%	-8.33%	7.37%	-0.16%	7.87%	-18.26%	11.56%	1.71%	-2.63%	0.77%	-11.97%	-6.65%	-11.52%	5.71%	9.57%	9.90%	17.18%	-12.29%	-19.42%	-0.74%	-0.80%	-2.54%	-18.31%	0.78%	-2.13%	10.14%
BRODMANN AREA 4 RIGHT	-8.59%	-0.48%	15.34%	3.48%	-0.10%	-16.44%	-4.57%	-7.23%	-3.38%	2.15%	-9.07%	-8.97%	-8.25%	8.12%	5.71%	7.01%	15.77%	-12.31%	-19.44%	0.50%	-1.20%	4.44%	-10.39%	4.89%	-1.79%	9.12%
BRODMANN AREA 5 LEFT	-16.20%	-2.58%	8.25%	-1.55%	2.34%	-18.00%	3.21%	-2.15%	-5.49%	6.68%	-18.26%	-13.98%	-19.24%	3.45%	2.05%	-3.98%	10.15%	0.75%	-14.63%	-0.48%	-0.21%	1.59%	-13.09%	13.64%	-3.24%	9.59%
BRODMANN AREA 5 RIGHT	-11.01%	0.56%	11.16%	10.06%	1.50%	-15.87%	2.98%	0.16%	-0.41%	-3.43%	-10.81%	-13.44%	-12.61%	4.11%	2.02%	6.54%	15.40%	-1.44%	-28.74%	10.91%	3.47%	5.12%	-11.89%	16.44%	-0.80%	10.96%
BRODMANN AREA 6 LEFT	-12.37%	-5.15%	13.00%	4.04%	2.57%	-15.38%	4.27%	0.30%	-5.53%	1.24%	-14.72%	-11.29%	-11.87%	1.97%	9.72%	3.71%	13.57%	-8.63%	-15.14%	-4.31%	-0.27%	2.76%	-13.03%	4.78%	-2.32%	8.93%
BRODMANN AREA 6 RIGHT	-11.37%	-6.02%	10.74%	7.42%	-1.30%	-16.79%	-1.89%	-4.40%	-2.09%	4.18%	-5.90%	-8.33%	-7.53%	4.46%	4.27%	5.86%	15.99%	-6.18%	-18.81%	1.81%	4.66%	3.55%	-14.28%	11.98%	-1.25%	9.07%
BRODMANN AREA 7 LEFT	-13.32%	1.99%	2.29%	-2.45%	-3.59%	-11.27%	0.86%	-3.85%	1.34%	14.45%	-9.42%	-11.70%	-10.93%	3.87%	-1.41%	0.43%	12.27%	5.21%	-16.92%	2.08%	6.89%	5.94%	-15.04%	17.74%	-1.02%	9.30%
BRODMANN AREA 7 RIGHT	-7.26%	1.08%	4.36%	8.51%	-2.20%	-13.45%	3.82%	3.03%	0.21%	6.94%	-7.78%	-7.99%	-10.18%	6.44%	-0.10%	5.06%	13.61%	3.69%	-22.61%	8.41%	1.83%	9.04%	-9.29%	17.21%	0.52%	9.19%
BRODMANN AREA 8 LEFT	-7.12%	-5.20%	3.56%	3.52%	-3.09%	-14.18%	0.14%	-1.96%	-6.19%	3.37%	-13.17%	-11.99%	-5.11%	2.44%	2.33%	7.10%	9.61%	4.31%	-14.16%	4.67%	6.79%	8.14%	-13.01%	-1.26%	-1.69%	7.58%
BRODMANN AREA 8 RIGHT	-6.20%	-6.28%	5.40%	2.09%	-4.68%	-9.15%	-0.28%	-7.37%	-4.36%	6.82%	-6.62%	-7.76%	-6.76%	8.35%	1.59%	6.29%	13.84%	-1.34%	-16.67%	3.00%	14.91%	0.59%	-13.42%	4.77%	-0.97%	8.03%
BRODMANN AREA 9 LEFT	-12.65%	-1.93%	10.41%	-3.48%	-3.20%	-14.49%	-1.20%	-4.93%	-7.82%	5.68%	-11.56%	-10.96%	-11.07%	-0.97%	5.38%	11.48%	8.39%	-1.21%	-14.33%	2.24%	12.74%	5.87%	-16.34%	4.83%	-2.05%	8.92%
BRODMANN AREA 9 RIGHT	-10.69%	-4.12%	7.46%	-1.72%	-0.85%	-14.98%	-4.81%	-0.89%	-8.02%	5.61%	-1.80%	-10.95%	-13.06%	4.16%	5.11%	4.18%	8.59%	5.77%	-15.30%	-0.26%	15.87%	2.74%	-14.14%	1.79%	-1.68%	8.41%
BRODMANN AREA 10 LEFT	-12.38%	-1.35%	8.76%	-2.69%	-2.27%	-19.31%	-2.20%	5.72%	-14.88%	8.68%	-15.90%	-9.01%	-13.50%	1.59%	8.92%	2.24%	3.76%	-1.30%	-17.52%	-1.28%	16.31%	-0.78%	-14.97%	4.49%	-2.87%	9.76%
BRODMANN AREA 10 RIGHT	-11.17%	-5.21%	4.70%	-3.07%	-2.30%	-16.99%	-5.65%	6.97%	-9.75%	0.68%	-11.99%	-14.83%	-11.80%	2.45%	3.18%	1.57%	5.32%	-0.51%	-20.18%	-2.15%	16.49%	-0.69%	-10.09%	8.19%	-3.20%	8.87%
BRODMANN AREA 11 LEFT	-0.73%	-1.89%	7.51%	-8.14%	-1.27%	-18.53%	0.20%	1.02%	-4.34%	13.01%	-17.08%	-15.26%	-18.58%	4.53%	4.75%	-0.07%	4.47%	-2.32%	-19.07%	-11.80%	18.49%	-0.83%	-13.81%	1.07%	-3.28%	10.17%
BRODMANN AREA 11 RIGHT	1.01%	-2.29%	5.53%	-1.48%	2.22%	-15.24%	-6.30%	4.69%	-7.55%	17.21%	-14.39%	-14.87%	-14.96%	2.91%	13.17%	2.58%	7.80%	-11.69%	-23.28%	-0.72%	15.66%	-1.58%	-11.47%	5.36%	-1.99%	10.60%
BRODMANN AREA 17 LEFT	-11.13%	4.12%	-2.97%	5.57%	3.22%	-19.45%	-3.68%	-4.14%	2.82%	12.37%	1.17%	-19.20%	-13.79%	1.10%	1.35%	0.29%	4.68%	-1.34%	-17.46%	-2.61%	1.94%	7.33%	-16.71%	11.84%	-2.28%	9.35%
BRODMANN AREA 17 RIGHT	5.91%	12.80%	1.32%	13.18%	-16.40%	-23.14%	3.11%	-2.52%	-2.59%	11.48%	-11.26%	-17.66%	-10.39%	2.52%	1.03%	1.08%	6.00%	2.43%	-28.91%	-0.44%	3.94%	15.03%	-2.67%	20.76%	-0.64%	12.22%
BRODMANN AREA 18 LEFT	-5.56%	5.95%	3.00%	2.77%	2.37%	-17.72%	-0.07%	-3.31%	6.56%	16.06%	-5.94%	-15.92%	-4.18%	-2.19%	2.02%	1.36%	7.07%	1.61%	-21.07%	-1.45%	2.81%	9.63%	-15.41%	10.59%	-0.88%	9.24%
BRODMANN AREA 18 RIGHT	3.93%	11.22%	6.19%	4.36%	-7.24%	-21.56%	4.61%	2.77%	-4.49%	3.06%	-7.88%	-15.27%	-14.79%	2.81%	-5.29%	6.95%	8.15%	5.59%	-25.79%	-1.38%	5.48%	9.57%	-6.65%	14.12%	-0.90%	10.39%
BRODMANN AREA 19 LEFT	-8.58%	-0.49%	1.31%	1.42%	-0.32%	-15.44%	1.39%	1.66%	1.27%	10.51%	-12.63%	-8.16%	-4.04%	-4.47%	0.99%	3.96%	10.39%	-0.40%	-18.74%	-4.51%	2.61%	7.34%	-11.98%	15.61%	-1.30%	8.34%
BRODMANN AREA 19 RIGHT	-3.57%	6.05%	2.94%	5.91%	2.65%	-19.98%	-2.85%	9.08%	0.30%	3.66%	-10.52%	-6.60%	-7.78%	4.65%	0.04%	8.18%	11.79%	-0.94%	-22.68%	-0.26%	4.81%	9.04%	-7.14%	7.80%	-0.23%	8.79%
BRODMANN AREA 20 LEFT	-4.32%	0.25%	3.37%	8.88%	8.50%	-15.30%	2.40%	4.29%	-6.49%	10.64%	-8.80%	-21.05%	-12.10%	-1.29%	8.97%	6.42%	4.95%	-10.00%	-15.39%	-8.69%	4.77%	4.24%	-12.87%	5.81%	-1.78%	9.29%
BRODMANN AREA 20 RIGHT	1.72%	5.73%	-0.88%	4.17%	-1.83%	-10.19%	1.62%	-3.97%	-4.13%	0.28%	-1.00%	-10.78%	-19.39%	-0.46%	7.89%	-9.05%	10.28%	-3.02%	-7.82%	-7.82%	2.83%	1.60%	-8.33%	-3.46%	-2.33%	6.70%
BRODMANN AREA 21 LEFT	-4.24%	-3.90%	5.48%	-7.05%	4.79%	-7.88%	7.71%	2.01%	-0.83%	14.70%	-16.42%	-15.68%	-12.47%	-2.75%	-1.13%	-1.81%	11.12%	-14.13%	-10.90%	-5.57%	14.73%	-6.24%	-13.98%	1.79%	-2.61%	9.15%
BRODMANN AREA 21 RIGHT	-1.02%	-1.56%	1.14%	-1.71%	-1.38%	-12.83%	-1.79%	-6.38%	-2.24%	8.20%	-4.76%	-11.61%	-22.39%	-2.72%	3.66%	-0.81%	5.54%	-3.91%	-10.66%	-5.30%	6.79%	5.86%	-6.62%	3.49%	-2.63%	7.01%
BRODMANN AREA 22 LEFT	-6.34%	-6.88%	2.29%	-3.59%	-0.16%	-18.18%	2.93%	8.14%	-0.01%	7.21%	-16.38%	-12.65%	-11.66%	-7.60%	8.88%	-2.35%	19.93%	-5.36%	-8.17%	-9.91%	4.46%	-4.13%	-14.03%	-1.57%	-3.13%	9.00%
BRODMANN AREA 22 RIGHT	-10.35%	-7.62%	1.27%	-6.51%	-1.11%	-19.59%	-4.72%	-4.02%	-0.25%	1.57%	-4.44%	-11.75%	-13.82%	-0.05%	6.32%	0.26%	15.76%	-1.69%	-14.89%	-11.74%	-2.72%	-3.68%	-9.27%	-1.93%	-4.37%	7.48%
BRODMANN AREA 23 LEFT	-19.77%	6.77%	-1.44%	7.76%	-0.07%	-24.74%	-1.88%	0.18%	-7.73%	2.37%	-17.47%	-4.47%	-11.13%	7.26%	3.59%	0.65%	6.67%	12.98%	-11.34%	5.34%	3.65%	-0.57%	-11.38%	17.10%	-1.57%	10.30%
BRODMANN AREA 23 RIGHT	-15.83%	5.02%	-0.51%	6.48%	3.54%	-24.98%	-4.86%	5.44%	-8.70%	-0.19%	-19.52%	-0.60%	-14.93%	4.99%	4.95%	1.27%	11.65%	17.20%	-15.57%	-0.93%	7.89%	-2.29%	-14.36%	14.60%	-1.68%	11.10%
BRODMANN AREA 24 LEFT	-14.52%	-2.59%	14.52%	-5.20%	-6.26%	-5.92%	2.34%	0.41%	6.07%	-0.40%	-20.61%	-4.89%	-14.99%	-7.77%	14.18%	2.29%	11.25%	4.03%	-11.34%	-8.08%	0.83%	-1.14%	-12.77%	-9.68%	-2.93%	9.09%
BRODMANN AREA 24 RIGHT	-9.01%	-0.96%	12.00%	-2.16%	-7.04%	-8.92%	2.47%	2.62%	-1.10%	-1.91%	-20.17%	-5.87%	-9.39%	-8.35%	11.27%	8.39%	13.09%	-5.57%	-17.22%	-3.43%	0.85%	3.67%	-16.12%	-7.75%	-2.94%	8.88%
BRODMANN AREA 25 LEFT	-10.76%	0.78%	7.23%	2.21%	-11.62%	-9.53%	-7.13%	-6.01%	6.11%	2.68%	-13.99%	-22.51%	-16.35%	-4.42%	8.00%	3.22%	9.92%	-2.76%	-9.97%	-12.01%	7.67%	-4.42%	-6.66%	-2.27%	-3.86%	8.63%
BRODMANN AREA 25 RIGHT	-10.63%	-5.03%	1.83%	1.12%	-3.64%	-16.12%	-4.22%	-7.53%	-6.90%	31.34%	0.07%	-10.56%	-2.90%	4.26%	8.76%	7.19%	17.16%	-13.96%	-18.44%	-6.81%	9.55%	4.31%	-11.57%	0.70%	-1.33%	11.16%
BRODMANN AREA 28 LEFT	-2.07%	-2.09%	3.22%	3.34%	1.35%	-18.33%	-3.62%	9.48%	-2.80%	8.75%	-6.71%	-14.41%	-7.11%	-0.61%	-2.62%	1.56%	7.45%	-12.27%	-19.19%	-4.72%	5.97%	2.44%	-17.78%	10.93%	-2.49%	8.83%
BRODMANN AREA 28 RIGHT	-10.47%	3.28%	0.23%	4.52%	7.16%	-5.13%	7.97%	-2.90%	0.24%	1.23%	-10.60%	-15.93%	-16.63%	5.85%	8.56%	-3.43%	5.47%	-2.00%	-11.49%	-4.80%	3.28%	2.77%	-5.16%	-4.48%	-1.77%	7.32%
BRODMANN AREA 31 LEFT	-13.75%	5.35%	-1.48%	3.99%	-10.41%	-16.68%	4.86%	-2.34%	-6.24%	5.68%	-11.27%	-7.94%	-10.88%	5.19%	0.48%	5.13%	6.75%	15.60%	-14.84%	7.10%	-1.33%	9.06%	-12.00%	14.47%	-1.06%	9.45%
BRODMANN AREA 31 RIGHT	-15.82%	9.24%	3.05%	3.49%	-5.67%	-19.04%	-2.19%	0.60%	-4.83%	1.75%	-12.44%	-3.19%	-9.33%	4.35%	-0.68%	17.12%	11.30%	13.41%	-19.72%	1.98%	-0.82%	-5.27%	-10.39%	10.48%	-1.36%	9.89%
BRODMANN AREA 32 LEFT	-12.38%	-3.29%	10.47%	-0.60%	2.50%	-17.11%	-4.17%	-3.13%	-7.64%	10.42%	-12.75%	-11.61%	-14.23%	6.47%	15.25%	-0.87%	5.20%	-4.20%	-19.16%	-8.28%	18.26%	2.91%	-12.93%	-2.14%	-2.63%	10.15%
BRODMANN AREA 32 RIGHT	-11.25%	-1.90%	6.39%	0.68%	0.46%	-19.21%	-4.95%	-4.60%	-6.15%	14.25%	-14.35%	-12.82%	-12.55%	4.53%	11.39%	0.66%	11.05%	0.59%	-20.65%	-5.33%	12.43%	5.65%	-16.88%	-6.71%	-2.89%	10.21%
BRODMANN AREA 36 LEFT	-7.61%	4.43%	0.78%	11.83%	-8.65%	-21.40%	-7.01%	-1.41%	-3.51%	20.44%	-18.13%	-16.48%	-4.52%	0.77%	5.81%	-1.42%	9.48%	-13.64%	-23.24%	-13.59%	5.79%	2.09%	-9.61%	18.31%	-2.94%	11.70%
BRODMANN AREA 36 RIGHT	-13.89%	3.75%	0.91%	13.13%	5.60%	-8.70%	-1.99%	-15.80%	-3.40%	-0.17%	-15.69%	-6.82%	-16.26%	-2.12%	12.34%	-8.91%	9.77%	-4.39%	-4.97%	-1.29%	2.98%	8.86%	1.75%	4.53%	-1.70%	8.67%
BRODMANN AREA 37 LEFT	-6.55%	1.36%	-2.85%	2.05%	3.66%	-17.32%	6.80%	11.67%	6.62%	4.76%	-17.03%	-25.74%	-19.57%	-7.65%	14.87%	-5.01%	-2.58%	-14.00%	-23.83%	-3.37%	0.99%	10.08%	-19.71%	14.37%	-3.67%	12.19%
BRODMANN AREA 37 RIGHT	-9.87%	7.21%	-4.59%	-0.02%	-3.35%	-18.67%	-11.25%	-0.06%	-5.66%	10.33%	-0.51%	-3.89%	-25.60%	3.28%	11.84%	3.65%	15.92%	4.15%	-16.90%	0.93%	10.64%	6.16%	-2.95%	15.30%	-0.58%	10.58%
BRODMANN AREA 38 LEFT	-1.53%	0.72%	8.04%	10.31%	7.81%	-13.86%	-0.93%	-2.81%	-1.56%	14.93%	-9.37%	-19.33%	-5.13%	2.84%	8.52%	-1.19%	5.56%	-12.60%	-12.76%	-0.21%	10.12%	-6.50%	-12.67%	-2.59%	-1.43%	8.96%
BRODMANN AREA 38 RIGHT	3.83%	-2.75%	0.41%	3.50%	7.95%	-19.01%	6.26%	-4.92%	-2.91%	-7.87%	0.62%	-13.24%	-17.29%	0.47%	7.22%	-6.09%	12.54%	-6.05%	-5.43%	-9.85%	4.34%	-2.93%	-7.58%	4.30%	-2.27%	7.96%
BRODMANN AREA 39 LEFT	-11.36%	6.87%	-0.74%	-1.98%	0.85%	-13.28%	7.37%	3.77%	-4.54%	7.01%	-20.75%	-13.12%	-11.67%	-2.59%	1.04%	-6.03%	12.28%	-1.71%	-10.37%	-6.20%	3.63%	8.27%	-16.72%	14.95%	-2.29%	9.41%
BRODMANN AREA 39 RIGHT	-3.07%	2.48%	2.56%	1.13%	-5.11%	-16.93%	3.85%	5.97%	-2.50%	-3.37%	-0.47%	-11.25%	-20.90%	-5.50%	-4.39%	0.67%	14.54%	-1.93%	-15.12%	2.66%	4.82%	9.75%	-13.38%	-1.87%	-2.39%	8.42%
BRODMANN AREA 40 LEFT	-11.88%	-5.18%	-0.64%	-7.88%	2.64%	-16.61%	-2.50%	8.14%	0.34%	5.75%	-14.62%	-15.08%	-8.71%	-1.10%	-1.59%	-2.45%	18.67%	-6.58%	-7.87%	-6.22%	6.62%	-1.14%	-16.68%	10.65%	-3.08%	8.96%
BRODMANN AREA 40 RIGHT	-3.97%	-1.07%	8.07%	-10.21%	-1.78%	-16.64%	3.09%	3.02%	2.92%	6.52%	3.53%	-9.03%	-15.85%	5.53%	6.34%	10.23%	14.80%	-1.70%	-20.47%	-3.99%	12.30%	4.48%	-18.07%	1.97%	-0.83%	9.78%
BRODMANN AREA 44 LEFT	-3.24%	-1.49%	11.83%	0.07%	4.88%	-15.61%	2.87%	1.24%	-4.95%	-0.90%	-16.30%	-14.08%	-19.57%	-6.90%	12.11%	-1.76%	5.82%	-9.93%	-7.41%	-8.51%	9.73%	0.22%	-10.79%	1.32%	-2.97%	8.75%
BRODMANN AREA 44 RIGHT	-10.77%	-0.59%	7.89%	-4.62%	4.19%	-18.43%	-4.48%	-1.40%	-3.86%	2.85%	-7.60%	-16.28%	-16.53%	2.80%	13.44%	2.34%	7.64%	-7.22%	-14.99%	0.14%	9.52%	-2.12%	-17.68%	7.06%	-2.86%	9.30%
BRODMANN AREA 45 LEFT	-7.31%	2.60%	10.05%	-8.99%	3.60%	-15.72%	-1.46%	0.32%	-13.81%	3.60%	-9.75%	-8.13%	-14.29%	-0.60%	6.25%	8.13%	10.74%	-11.57%	-9.39%	-8.24%	12.47%	-4.50%	-15.17%	-4.46%	-3.15%	8.78%
BRODMANN AREA 45 RIGHT	-6.16%	-8.05%	10.50%	-11.57%	9.02%	-21.15%	-0.69%	6.32%	-5.00%	-7.74%	-11.71%	-15.57%	-13.22%	4.35%	10.85%	4.70%	4.41%	-6.78%	-3.43%	3.49%	18.88%	-1.46%	-12.85%	-0.32%	-2.22%	9.80%
BRODMANN AREA 46 LEFT	-8.83%	-4.85%	11.01%	-4.04%	-2.94%	-5.13%	-9.00%	-0.46%	-5.49%	-1.32%	-14.09%	-7.28%	-10.23%	-3.93%	13.17%	-3.15%	6.68%	-8.67%	-15.95%	-1.51%	8.36%	2.73%	-15.46%	2.93%	-3.23%	7.78%
BRODMANN AREA 46 RIGHT	-3.25%	-12.13%	10.51%	-7.84%	-1.57%	-20.78%	-8.85%	-3.74%	-7.66%	4.26%	-7.43%	-6.66%	-22.73%	-3.74%	8.74%	1.40%	-0.64%	-5.59%	-15.33%	-3.13%	19.95%	5.17%	-9.61%	-5.17%	-3.99%	9.51%
BRODMANN AREA 47 LEFT	-10.90%	-7.71%	7.35%	-6.32%	9.20%	-19.45%	1.79%	12.69%	-4.45%	-16.05%	-17.35%	-14.45%	-14.06%	-3.04%	10.64%	-0.32%	3.00%	3.43%	-15.90%	-9.04%	13.98%	-3.76%	-13.32%	3.46%	-3.77%	10.26%
BRODMANN AREA 47 RIGHT	1.60%	-3.55%	7.30%	-4.32%	7.13%	-20.72%	4.01%	-0.82%	-3.74%	-3.07%	-14.74%	-17.04%	-21.61%	-1.39%	13.94%	-5.77%	0.04%	-11.09%	-14.20%	-11.32%	16.03%	4.56%	-17.27%	-5.40%	-4.23%	10.30%

Table S1-2. The relative change percentage of SPECT imaging data for patients in the control group after HBOT, in each Brodmann area.

	subject #																									
	1	2	3	4	5	6	7	8	9	10	11	12	13	14	15	16	17	18	19	20	21	22	23	24	**AVG**	**SD**
BRODMANN AREA 4 LEFT	12.31%	2.99%	-2.53%	-3.08%	-10.67%	-1.43%	3.08%	-9.09%	-2.63%	-9.59%	7.94%	8.93%	-4.92%	6.45%	-1.39%	-8.02%	5.45%	3.23%	36.84%	10.81%	-3.08%	6.59%	-1.54%	-7.69%	1.62%	10.05%
BRODMANN AREA 4 RIGHT	3.08%	0.00%	-7.69%	-7.58%	0.00%	7.35%	15.79%	-4.69%	-3.38%	-6.67%	-2.99%	16.36%	1.59%	9.52%	5.97%	-6.33%	1.82%	13.56%	36.84%	13.89%	1.56%	2.65%	-10.14%	-6.35%	3.09%	10.66%
BRODMANN AREA 5 LEFT	0.00%	-7.25%	-2.78%	-8.82%	-8.82%	1.43%	10.71%	1.56%	-5.49%	-11.27%	-3.08%	14.81%	8.62%	4.41%	-5.48%	-3.18%	1.72%	-1.54%	17.54%	2.74%	-1.56%	7.07%	-6.56%	-15.15%	-0.43%	8.07%
BRODMANN AREA 5 RIGHT	-1.79%	-8.11%	-7.04%	-10.45%	5.88%	4.84%	4.84%	-9.38%	-0.41%	-3.13%	-1.52%	20.41%	1.67%	11.48%	1.61%	-4.50%	-1.89%	8.20%	41.18%	7.04%	-3.13%	-4.32%	-8.62%	-18.84%	1.00%	11.86%
BRODMANN AREA 6 LEFT	7.46%	2.86%	-7.14%	-8.82%	-2.74%	-7.04%	12.70%	-1.56%	-5.53%	-9.33%	0.00%	10.91%	3.17%	8.33%	-2.70%	-0.19%	5.36%	8.33%	32.26%	9.21%	2.99%	2.96%	-4.48%	-5.88%	2.13%	9.15%
BRODMANN AREA 6 RIGHT	4.48%	5.71%	-4.88%	-11.27%	2.90%	2.94%	14.75%	-1.52%	-2.09%	-8.00%	-1.47%	8.93%	0.00%	8.06%	4.23%	-2.25%	3.51%	8.33%	37.29%	3.80%	-1.47%	0.87%	-10.29%	-4.35%	2.43%	9.67%
BRODMANN AREA 7 LEFT	6.67%	-8.33%	-3.90%	-10.00%	0.00%	-2.70%	16.13%	-1.52%	1.34%	-18.18%	0.00%	6.90%	3.23%	4.29%	-2.60%	-0.72%	-1.47%	1.45%	23.73%	2.70%	-7.25%	1.51%	0.00%	-20.83%	-0.40%	9.27%
BRODMANN AREA 7 RIGHT	1.64%	1.35%	-5.19%	-10.96%	11.27%	5.97%	10.29%	-11.11%	0.21%	-11.43%	4.29%	5.56%	6.67%	8.96%	1.52%	-3.15%	-1.67%	4.55%	33.93%	2.70%	-5.97%	-3.29%	-6.35%	-19.44%	0.85%	10.40%
BRODMANN AREA 8 LEFT	1.47%	-1.33%	-2.41%	-10.00%	9.46%	-9.86%	15.63%	4.69%	-6.19%	-10.26%	1.49%	12.96%	-1.49%	1.59%	0.00%	-5.13%	3.45%	-3.13%	34.92%	2.38%	-6.85%	-3.15%	-5.71%	-5.97%	0.69%	9.93%
BRODMANN AREA 8 RIGHT	1.43%	3.95%	1.18%	-11.59%	2.74%	-1.37%	14.06%	8.06%	-4.36%	-17.50%	-1.45%	12.73%	4.55%	1.52%	0.00%	-5.39%	1.69%	6.45%	35.48%	3.70%	-11.84%	5.51%	-8.70%	-5.88%	1.46%	10.46%
BRODMANN AREA 9 LEFT	2.90%	-3.85%	-1.12%	-4.41%	2.67%	-5.71%	21.54%	0.00%	-7.82%	-12.35%	4.48%	7.02%	2.99%	4.84%	0.00%	-6.31%	0.00%	4.69%	31.34%	2.44%	-11.84%	4.93%	-5.56%	-5.97%	1.04%	9.60%
BRODMANN AREA 9 RIGHT	2.90%	-3.90%	-2.30%	-5.71%	4.05%	1.41%	20.63%	-1.47%	-8.02%	-16.87%	-4.23%	10.91%	14.06%	4.69%	-1.25%	-6.78%	5.08%	1.54%	29.23%	4.88%	-12.00%	2.03%	-4.11%	-4.48%	1.26%	10.03%
BRODMANN AREA 10 LEFT	-1.43%	-2.56%	-3.33%	-2.99%	4.11%	0.00%	16.42%	-4.23%	-14.88%	-10.98%	6.15%	5.00%	5.88%	3.03%	-3.66%	-2.27%	1.67%	11.29%	33.85%	0.00%	-14.47%	5.18%	-2.63%	0.00%	1.21%	9.96%
BRODMANN AREA 10 RIGHT	0.00%	-5.06%	-2.25%	-8.70%	1.35%	-1.41%	17.65%	-5.48%	-9.75%	-12.05%	1.52%	12.28%	13.64%	4.62%	-7.14%	-2.33%	3.33%	6.35%	39.06%	1.16%	-10.53%	0.04%	-7.59%	-5.56%	0.96%	11.16%
BRODMANN AREA 11 LEFT	3.08%	0.00%	-1.27%	-6.78%	-1.49%	6.06%	12.70%	3.03%	-4.34%	-6.76%	8.20%	3.57%	10.00%	5.00%	-9.21%	-2.25%	7.02%	6.90%	44.64%	11.27%	-9.72%	4.82%	-4.29%	4.84%	3.54%	10.84%
BRODMANN AREA 11 RIGHT	0.00%	7.14%	-1.25%	-3.17%	0.00%	4.41%	7.69%	0.00%	-7.55%	-11.25%	1.59%	7.14%	10.61%	10.00%	-6.17%	0.00%	0.00%	15.00%	40.00%	1.27%	-13.75%	-2.63%	-9.86%	-6.35%	1.79%	10.86%
BRODMANN AREA 17 LEFT	-8.45%	-4.23%	0.00%	-17.98%	2.50%	10.00%	17.39%	-1.15%	2.82%	-10.34%	-5.68%	13.70%	19.44%	2.27%	-4.21%	-2.67%	-5.75%	-3.90%	18.18%	9.46%	0.00%	1.30%	-1.59%	-21.05%	0.42%	10.38%
BRODMANN AREA 17 RIGHT	-10.53%	-11.11%	-2.33%	-9.21%	16.00%	21.43%	10.00%	-10.13%	-2.59%	-8.97%	2.53%	6.35%	9.38%	9.21%	0.00%	-1.39%	-2.82%	-5.33%	40.35%	5.13%	3.85%	-8.72%	-3.28%	-25.30%	0.94%	13.13%
BRODMANN AREA 18 LEFT	-7.14%	-5.41%	0.00%	-11.69%	2.63%	9.21%	11.76%	-2.63%	6.56%	-14.63%	0.00%	1.52%	7.35%	6.76%	0.00%	-1.40%	-2.74%	-2.99%	26.23%	6.25%	-4.05%	-2.63%	1.49%	-18.18%	0.26%	9.12%
BRODMANN AREA 18 RIGHT	-7.04%	-6.17%	-3.57%	-6.33%	4.17%	20.90%	7.04%	-12.05%	-4.49%	-11.69%	1.30%	3.23%	12.90%	5.48%	6.94%	-4.10%	2.90%	-2.99%	28.81%	8.00%	-2.63%	-4.82%	-5.97%	-20.51%	0.39%	10.66%
BRODMANN AREA 19 LEFT	-1.52%	-4.35%	-2.60%	-6.94%	4.35%	0.00%	10.61%	-10.39%	1.27%	-14.47%	1.43%	0.00%	9.23%	11.27%	-3.85%	-0.75%	-1.41%	-2.99%	19.67%	8.11%	-6.67%	6.06%	0.00%	-18.67%	-0.11%	8.47%
BRODMANN AREA 19 RIGHT	-4.62%	-2.74%	-5.26%	-2.78%	5.48%	12.50%	8.96%	-12.99%	0.30%	-9.59%	4.41%	3.57%	3.28%	10.45%	0.00%	-2.69%	-1.56%	3.13%	35.19%	5.26%	-2.74%	-0.88%	-7.58%	-20.55%	0.77%	10.43%
BRODMANN AREA 20 LEFT	8.20%	-2.94%	3.90%	-8.20%	2.90%	6.90%	9.84%	-2.99%	-6.49%	-8.22%	-7.94%	14.58%	10.00%	12.73%	-1.39%	1.44%	-5.45%	9.43%	29.63%	15.28%	-3.17%	1.09%	-5.97%	-4.76%	2.85%	9.50%
BRODMANN AREA 20 RIGHT	1.54%	-9.72%	8.33%	-16.13%	1.61%	4.92%	1.69%	1.54%	-4.13%	-17.14%	0.00%	14.00%	11.86%	5.36%	-8.22%	5.97%	1.82%	5.56%	21.43%	9.72%	-8.06%	2.65%	-1.52%	-6.56%	1.11%	9.12%
BRODMANN AREA 21 LEFT	-4.41%	4.05%	5.13%	-5.88%	7.04%	0.00%	0.00%	-12.33%	-0.83%	-4.05%	1.59%	3.64%	10.94%	7.69%	-4.00%	3.96%	0.00%	8.33%	22.22%	6.58%	-5.48%	8.44%	0.00%	0.00%	2.19%	7.02%
BRODMANN AREA 21 RIGHT	-7.14%	1.32%	1.27%	-14.29%	7.35%	14.06%	9.52%	4.41%	-2.24%	-13.70%	1.43%	7.41%	19.30%	1.52%	-8.97%	1.24%	11.67%	6.67%	24.14%	12.16%	-1.45%	-2.37%	-6.85%	-8.57%	2.41%	9.84%
BRODMANN AREA 22 LEFT	0.00%	1.35%	2.35%	-5.88%	5.48%	15.38%	2.78%	-10.67%	-0.01%	-10.13%	4.41%	6.90%	10.61%	10.94%	-3.75%	8.56%	-11.43%	8.06%	23.19%	3.85%	-4.17%	3.08%	-5.19%	-5.63%	2.09%	8.47%
BRODMANN AREA 22 RIGHT	1.41%	-5.19%	-3.49%	-7.58%	2.74%	24.24%	15.15%	-8.00%	-0.25%	-12.66%	-4.11%	10.71%	14.06%	4.41%	-1.23%	1.06%	-4.48%	6.67%	45.90%	7.89%	1.41%	6.83%	-6.41%	-2.70%	3.60%	12.37%
BRODMANN AREA 23 LEFT	9.26%	-7.27%	7.94%	-3.03%	9.84%	-1.79%	12.50%	-1.89%	-7.73%	-14.29%	13.04%	-1.92%	2.04%	8.47%	4.48%	0.21%	8.89%	1.92%	32.73%	0.00%	-6.15%	3.58%	-13.33%	-20.00%	1.56%	10.89%
BRODMANN AREA 23 RIGHT	10.34%	0.00%	7.69%	-2.99%	7.58%	-3.57%	15.25%	-12.96%	-8.70%	-13.11%	19.15%	-5.66%	4.08%	7.14%	6.15%	2.69%	6.67%	-3.77%	39.62%	2.99%	-10.14%	5.00%	-11.67%	-16.67%	1.88%	12.34%
BRODMANN AREA 24 LEFT	0.00%	-1.72%	-12.16%	8.00%	18.18%	-10.20%	24.14%	0.00%	6.07%	-9.26%	10.42%	4.08%	8.00%	10.00%	-7.81%	1.77%	-16.00%	15.22%	27.27%	3.45%	-3.70%	2.01%	0.00%	7.69%	3.56%	10.88%
BRODMANN AREA 24 RIGHT	1.64%	0.00%	-12.99%	8.00%	21.28%	-8.00%	12.31%	-13.56%	-1.10%	-15.00%	8.51%	0.00%	0.00%	12.96%	-9.52%	-0.47%	-3.77%	10.64%	32.76%	6.25%	-5.36%	-2.02%	0.00%	1.79%	1.85%	11.11%
BRODMANN AREA 25 LEFT	0.00%	-14.10%	-7.87%	-8.82%	1.54%	4.00%	15.63%	2.86%	6.11%	-9.33%	-1.43%	14.04%	18.03%	5.97%	-5.06%	-5.05%	3.03%	0.00%	17.65%	15.58%	-5.00%	6.36%	4.94%	0.00%	2.46%	8.97%
BRODMANN AREA 25 RIGHT	1.37%	-1.41%	-2.27%	-13.85%	1.49%	13.43%	13.43%	-4.29%	-6.90%	-27.06%	-14.10%	10.53%	0.00%	4.41%	-10.39%	0.58%	-3.03%	16.39%	26.56%	10.39%	-8.64%	6.44%	2.78%	16.13%	1.33%	11.85%
BRODMANN AREA 28 LEFT	0.00%	1.69%	1.61%	-3.92%	3.64%	3.64%	7.84%	-10.00%	-2.80%	-13.33%	0.00%	10.87%	1.92%	9.26%	3.39%	9.60%	4.00%	8.51%	32.61%	20.34%	-8.47%	1.21%	-1.79%	5.36%	3.55%	9.50%
BRODMANN AREA 28 RIGHT	1.89%	-6.25%	-1.64%	0.00%	1.79%	1.72%	-1.85%	-3.70%	0.24%	-18.03%	13.21%	13.33%	12.77%	3.70%	-5.17%	-4.15%	13.04%	6.52%	20.00%	20.00%	-6.67%	5.64%	-11.29%	-5.66%	2.06%	9.59%
BRODMANN AREA 31 LEFT	1.61%	-6.76%	7.79%	-8.11%	0.00%	-2.56%	11.76%	-2.74%	-6.24%	-7.59%	1.47%	3.28%	-2.94%	14.08%	3.61%	-14.73%	-1.49%	5.71%	20.31%	-3.66%	-8.22%	-1.55%	-8.33%	-23.38%	-1.19%	9.29%
BRODMANN AREA 31 RIGHT	3.17%	-4.94%	3.70%	-6.76%	4.00%	-5.41%	14.29%	-8.45%	-4.83%	-2.67%	8.82%	1.69%	-1.47%	7.25%	2.60%	-8.78%	-1.59%	7.46%	27.87%	3.75%	-5.41%	-2.17%	-6.94%	-23.38%	0.08%	9.65%
BRODMANN AREA 32 LEFT	3.28%	1.49%	-2.41%	-1.61%	1.61%	-4.69%	14.29%	-3.17%	-7.64%	-12.00%	6.45%	7.41%	9.52%	7.94%	-7.79%	0.58%	-3.51%	7.41%	27.87%	5.19%	-12.33%	-1.12%	-2.86%	-1.61%	1.35%	8.76%
BRODMANN AREA 32 RIGHT	4.84%	3.03%	-1.20%	-3.23%	-1.59%	1.61%	12.70%	-1.67%	-6.15%	-13.92%	-1.56%	11.32%	4.62%	9.38%	-9.21%	-1.41%	0.00%	5.45%	37.10%	4.82%	-12.33%	-0.88%	-2.82%	-6.25%	1.36%	10.12%
BRODMANN AREA 36 LEFT	3.77%	-1.69%	-1.59%	-7.69%	5.66%	5.77%	12.00%	0.00%	-3.51%	-16.67%	7.84%	22.22%	0.00%	14.81%	0.00%	9.33%	-4.00%	6.82%	21.28%	35.71%	-1.69%	-1.66%	-8.20%	3.57%	4.25%	11.19%
BRODMANN AREA 36 RIGHT	9.26%	-1.59%	3.23%	-13.46%	1.82%	5.26%	3.92%	12.50%	-3.40%	-14.04%	19.61%	13.04%	19.57%	12.00%	-9.68%	-0.98%	8.70%	8.70%	15.09%	15.63%	-8.06%	-5.46%	-19.05%	-8.62%	2.67%	11.14%
BRODMANN AREA 37 LEFT	6.56%	-5.56%	13.51%	-11.11%	17.65%	0.00%	9.23%	-7.69%	6.62%	0.00%	0.00%	12.50%	20.97%	12.90%	-12.20%	2.01%	15.87%	3.08%	28.07%	-1.25%	-14.47%	8.15%	9.38%	-21.74%	3.85%	12.02%
BRODMANN AREA 37 RIGHT	0.00%	-1.37%	-1.41%	-4.62%	5.88%	6.90%	13.56%	-11.76%	-5.66%	-23.29%	1.37%	-1.89%	24.00%	16.67%	-11.69%	10.19%	8.06%	-3.03%	29.09%	1.33%	-2.94%	4.45%	-10.77%	-24.66%	0.77%	12.79%
BRODMANN AREA 38 LEFT	5.00%	4.84%	1.30%	-11.67%	-1.61%	10.34%	13.33%	-6.56%	-1.56%	-15.15%	1.67%	10.87%	5.26%	5.36%	-1.52%	1.56%	-7.27%	10.20%	36.36%	5.48%	-4.48%	0.83%	1.56%	5.08%	2.88%	9.98%
BRODMANN AREA 38 RIGHT	1.61%	-4.55%	10.00%	-10.17%	-4.84%	5.08%	6.67%	1.59%	-2.91%	-9.68%	1.61%	20.00%	5.45%	5.45%	-4.48%	9.90%	-1.82%	5.88%	17.54%	14.06%	-1.67%	4.34%	-4.76%	-8.20%	2.34%	8.15%
BRODMANN AREA 39 LEFT	1.45%	-11.54%	3.95%	-9.46%	2.74%	5.63%	7.14%	-6.67%	-4.54%	-12.99%	7.46%	5.08%	12.31%	2.82%	0.00%	7.82%	-5.41%	4.35%	8.96%	2.67%	-1.39%	2.73%	0.00%	-15.07%	0.34%	7.33%
BRODMANN AREA 39 RIGHT	-4.35%	-6.33%	-4.82%	-10.39%	11.27%	6.25%	17.14%	-14.47%	-2.50%	-12.50%	-5.06%	9.09%	14.75%	15.38%	5.80%	4.39%	1.52%	8.06%	33.33%	3.80%	-6.85%	-1.01%	-3.03%	-5.71%	2.24%	11.03%
BRODMANN AREA 40 LEFT	1.49%	-2.78%	-1.22%	-4.55%	5.71%	11.94%	22.22%	-12.16%	0.34%	-15.38%	4.17%	18.18%	-3.13%	4.48%	-2.63%	2.40%	-9.59%	9.84%	18.18%	16.67%	-4.17%	4.60%	1.47%	-18.92%	1.97%	10.59%
BRODMANN AREA 40 RIGHT	-5.71%	-9.88%	-8.05%	-4.55%	2.82%	7.58%	7.46%	-9.46%	2.92%	-22.22%	-1.32%	9.43%	11.86%	8.96%	1.32%	-3.67%	3.13%	13.56%	35.71%	5.19%	-8.00%	-1.91%	-10.29%	-2.86%	0.92%	11.25%
BRODMANN AREA 44 LEFT	-2.63%	-3.70%	-5.10%	-4.17%	3.90%	1.35%	14.08%	-2.74%	-4.95%	-3.80%	2.74%	6.67%	11.94%	11.29%	-7.14%	5.14%	6.25%	6.35%	14.86%	4.49%	-7.14%	5.17%	-5.13%	-5.63%	1.75%	6.94%
BRODMANN AREA 44 RIGHT	1.35%	1.28%	-2.20%	-6.94%	2.63%	4.17%	13.04%	-4.00%	-3.86%	-19.10%	0.00%	11.86%	13.43%	3.03%	-2.35%	-2.80%	6.25%	11.11%	28.99%	-5.43%	-2.44%	2.56%	-5.41%	-2.78%	1.77%	9.32%
BRODMANN AREA 45 LEFT	-5.56%	-2.44%	-1.08%	-6.85%	4.82%	2.70%	12.68%	-5.48%	-13.81%	-3.80%	1.37%	1.64%	14.29%	18.33%	1.18%	3.79%	6.45%	6.15%	29.58%	0.00%	-9.64%	18.09%	-1.23%	-1.41%	2.91%	9.88%
BRODMANN AREA 45 RIGHT	3.95%	7.79%	-3.26%	0.00%	-2.44%	7.25%	18.84%	-8.64%	-5.00%	-3.61%	10.96%	10.00%	9.86%	1.56%	-2.30%	-4.16%	9.84%	10.45%	29.87%	-2.25%	-10.23%	2.61%	-5.00%	8.33%	3.52%	9.25%
BRODMANN AREA 46 LEFT	2.63%	-3.75%	7.07%	-2.90%	7.79%	-7.69%	17.39%	-5.48%	-5.49%	-9.20%	5.33%	6.67%	1.37%	18.03%	-7.45%	2.57%	0.00%	4.48%	43.48%	5.62%	-15.66%	6.90%	0.00%	-6.76%	2.71%	11.82%
BRODMANN AREA 46 RIGHT	-8.43%	2.50%	0.00%	-11.27%	6.85%	12.86%	20.31%	-1.43%	-7.66%	-12.50%	-3.85%	7.02%	24.24%	4.41%	-7.61%	2.94%	14.75%	17.65%	29.85%	0.00%	-13.58%	6.17%	-13.33%	0.00%	2.91%	12.16%
BRODMANN AREA 47 LEFT	0.00%	-2.53%	7.61%	-5.71%	-3.66%	9.72%	6.67%	-12.50%	-4.45%	10.29%	1.39%	12.07%	15.15%	11.48%	-11.49%	3.52%	12.50%	-5.97%	37.88%	4.94%	-7.32%	11.30%	0.00%	-10.14%	3.36%	11.13%
BRODMANN AREA 47 RIGHT	-4.11%	2.47%	6.38%	-6.85%	1.28%	12.86%	0.00%	0.00%	-3.74%	-10.84%	-1.41%	10.34%	17.46%	4.62%	-5.95%	3.39%	18.03%	29.51%	33.33%	8.54%	-6.17%	2.31%	-4.11%	3.03%	4.60%	11.10%

Table S1-3. The relative change percentage of SPECT imaging data for patients in the treated group after HBOT, in each Brodmann area.

	subject #																															
	1	2	3	4	5	6	7	8	9	10	11	12	13	14	15	16	17	18	19	20	21	22	23	24	25	26	27	28	29	30	31	**AVG**	**SD**
BRODMANN AREA 4 LEFT	7.97%	4.85%	-3.25%	-7.14%	0.17%	-9.62%	4.84%	0.89%	-18.09%	1.44%	-1.69%	9.11%	4.19%	-5.25%	6.96%	-9.20%	-2.82%	6.84%	-11.93%	1.31%	-11.93%	1.68%	-11.26%	-2.69%	2.39%	1.77%	20.04%	6.05%	-6.08%	1.14%	9.53%	-0.32%	7.98%
BRODMANN AREA 4 RIGHT	14.70%	-3.31%	-6.67%	-1.93%	4.38%	-16.49%	3.69%	-1.49%	-21.38%	-0.21%	4.31%	6.11%	-4.47%	3.79%	9.30%	-11.17%	-3.65%	13.58%	-13.03%	-1.20%	-13.03%	4.90%	-4.72%	-3.60%	-12.38%	-4.34%	20.90%	-7.90%	-1.35%	0.09%	4.57%	-1.35%	9.27%
BRODMANN AREA 5 LEFT	8.51%	0.94%	-10.41%	-6.65%	-3.75%	-6.93%	13.78%	-0.45%	-21.17%	8.25%	-4.87%	16.03%	0.89%	-11.55%	-6.72%	-5.38%	-1.74%	5.29%	-8.00%	0.98%	-8.00%	-3.48%	-0.21%	-3.00%	5.56%	4.00%	24.39%	7.97%	-11.14%	-3.28%	-2.67%	-0.74%	9.12%
BRODMANN AREA 5 RIGHT	-0.71%	-0.68%	-1.63%	-10.52%	-8.44%	-17.13%	4.62%	-3.07%	-11.39%	11.03%	-2.99%	15.81%	1.19%	-3.18%	2.00%	-1.62%	-2.60%	8.45%	-10.90%	-1.90%	-10.90%	-3.94%	-0.69%	-0.31%	-9.06%	7.65%	21.33%	-0.78%	-12.42%	-5.51%	5.09%	-1.39%	8.41%
BRODMANN AREA 6 LEFT	7.45%	-0.79%	-3.02%	-1.84%	9.22%	-7.45%	4.25%	0.11%	-21.20%	-0.92%	5.53%	7.03%	4.87%	0.54%	5.90%	-12.17%	-1.62%	4.60%	-14.85%	3.18%	-14.85%	-4.93%	-7.11%	0.14%	-1.77%	7.18%	24.32%	1.65%	-4.09%	-0.90%	7.46%	-0.13%	8.63%
BRODMANN AREA 6 RIGHT	9.34%	0.37%	-3.73%	-1.63%	8.47%	-11.50%	9.06%	-0.55%	-20.49%	-0.93%	2.19%	11.03%	3.31%	0.43%	6.00%	-3.46%	-4.62%	7.82%	-14.28%	-2.13%	-14.28%	2.07%	-5.96%	0.85%	-7.01%	1.45%	16.65%	-3.03%	-5.88%	-5.50%	2.54%	-0.75%	8.01%
BRODMANN AREA 7 LEFT	11.76%	0.56%	-11.57%	1.62%	-0.06%	-11.27%	6.78%	-4.89%	-12.21%	2.36%	0.12%	19.05%	-1.97%	-13.14%	-7.22%	-11.63%	-0.69%	8.39%	-7.61%	1.17%	-7.61%	1.51%	1.44%	-0.17%	2.92%	3.11%	12.62%	6.12%	-12.70%	-3.44%	2.85%	-0.77%	8.01%
BRODMANN AREA 7 RIGHT	6.32%	0.70%	-11.66%	-3.88%	-0.74%	-15.29%	9.52%	-1.30%	-12.40%	12.52%	-0.95%	13.26%	-2.54%	-2.93%	2.54%	-10.76%	-4.61%	8.15%	-13.89%	-3.08%	-13.89%	-3.20%	4.03%	1.02%	-5.23%	4.33%	14.79%	1.90%	-12.25%	-3.10%	5.36%	-1.20%	8.30%
BRODMANN AREA 8 LEFT	9.33%	-3.41%	-5.74%	-1.48%	11.10%	-10.80%	6.67%	-2.65%	-23.07%	-0.46%	8.85%	11.52%	-1.37%	-4.66%	5.12%	-8.27%	-0.97%	8.02%	-12.09%	2.62%	-12.09%	-6.15%	-7.59%	2.17%	4.04%	2.74%	16.19%	1.67%	-7.46%	-0.41%	3.96%	-0.47%	8.38%
BRODMANN AREA 8 RIGHT	16.49%	2.31%	-3.97%	-0.15%	10.77%	-8.80%	12.03%	-4.72%	-22.95%	-0.17%	5.07%	12.02%	-1.22%	-1.25%	5.52%	-9.01%	0.65%	10.71%	-12.47%	-2.22%	-12.47%	-7.57%	-8.77%	3.32%	-6.70%	6.18%	15.91%	-1.14%	-1.57%	-7.17%	0.01%	-0.37%	9.00%
BRODMANN AREA 9 LEFT	9.51%	5.49%	-3.03%	-4.00%	8.52%	-12.08%	4.08%	-5.98%	-22.47%	-0.36%	-2.98%	10.57%	-4.23%	-5.93%	3.98%	-8.72%	3.06%	7.87%	-10.93%	3.98%	-10.93%	-6.51%	-10.63%	0.45%	2.26%	5.19%	11.67%	0.27%	-8.47%	-5.93%	1.24%	-1.45%	7.92%
BRODMANN AREA 9 RIGHT	18.84%	6.53%	-1.94%	4.84%	10.62%	-10.75%	7.00%	-3.42%	-20.47%	9.34%	5.04%	11.43%	-2.30%	-1.25%	6.70%	-14.69%	1.97%	9.59%	-10.61%	0.79%	-10.61%	0.96%	-11.62%	3.57%	-12.16%	5.51%	17.41%	-0.87%	-11.15%	-1.72%	-1.27%	0.17%	9.53%
BRODMANN AREA 10 LEFT	6.14%	6.80%	-1.72%	-1.45%	6.51%	-10.95%	6.64%	5.60%	-20.02%	1.23%	6.73%	9.71%	1.76%	-8.27%	11.01%	-9.11%	-1.62%	12.53%	-10.89%	4.02%	-10.89%	-6.15%	-4.32%	4.13%	-7.22%	8.71%	23.98%	3.15%	-6.47%	-4.15%	8.02%	0.76%	9.04%
BRODMANN AREA 10 RIGHT	8.69%	5.77%	-3.89%	-4.75%	7.03%	-4.76%	7.72%	-3.79%	-18.73%	1.09%	6.06%	9.40%	0.08%	-3.52%	6.32%	-13.72%	-2.56%	9.02%	-7.95%	6.28%	-7.95%	-6.18%	-5.54%	8.16%	-6.20%	11.36%	12.19%	-2.05%	-3.55%	-3.52%	4.61%	0.17%	7.67%
BRODMANN AREA 11 LEFT	6.06%	7.58%	-10.20%	1.28%	23.80%	-3.23%	2.10%	-3.27%	-22.27%	-4.15%	9.71%	6.58%	0.62%	-3.96%	2.30%	-14.21%	-4.86%	9.89%	-5.79%	3.87%	-5.79%	-3.61%	-3.07%	-1.65%	-1.39%	14.50%	30.53%	2.34%	-5.27%	8.52%	8.36%	1.46%	10.25%
BRODMANN AREA 11 RIGHT	2.19%	3.07%	-7.40%	-6.89%	20.15%	-4.02%	9.63%	10.07%	-25.33%	5.58%	-1.22%	5.42%	-1.93%	-1.08%	18.96%	-8.70%	-8.98%	11.62%	-4.37%	2.90%	-4.37%	-10.24%	-3.06%	-4.31%	3.95%	19.94%	12.92%	-2.27%	1.15%	-0.58%	8.87%	1.34%	9.80%
BRODMANN AREA 17 LEFT	11.33%	-1.59%	-11.27%	-6.74%	10.81%	-9.22%	5.79%	14.99%	-13.06%	5.42%	-9.60%	30.73%	-9.89%	-3.60%	-7.40%	-7.87%	-2.38%	-3.86%	-8.94%	-3.89%	-8.94%	15.28%	1.86%	-1.52%	18.15%	0.30%	10.74%	8.34%	-4.61%	-8.43%	2.84%	0.44%	10.44%
BRODMANN AREA 17 RIGHT	16.23%	-13.95%	-18.81%	-14.98%	7.76%	-15.26%	2.25%	9.91%	-13.83%	11.46%	-5.28%	16.88%	-16.85%	9.26%	-8.82%	-15.53%	-6.44%	7.43%	-6.90%	-8.67%	-6.90%	8.99%	-2.25%	-3.06%	-8.73%	3.14%	17.92%	1.79%	-3.98%	-6.30%	-3.47%	-2.16%	10.73%
BRODMANN AREA 18 LEFT	14.14%	-1.21%	-12.74%	-3.67%	8.72%	-10.26%	1.80%	10.83%	-11.75%	7.90%	-7.95%	14.04%	-8.26%	-8.54%	-8.75%	-12.75%	-2.16%	1.35%	-8.16%	0.50%	-8.16%	7.64%	1.86%	1.41%	18.26%	-6.56%	6.30%	6.75%	-7.31%	-7.36%	-0.14%	-0.78%	8.71%
BRODMANN AREA 18 RIGHT	14.23%	-10.81%	-11.28%	-9.85%	9.37%	-10.17%	4.53%	4.52%	-12.41%	10.20%	-5.30%	16.90%	-13.41%	8.40%	-8.49%	-15.34%	-8.53%	7.13%	-9.08%	-2.28%	-9.08%	8.38%	3.34%	-6.63%	2.19%	-1.31%	10.01%	1.13%	-3.18%	-5.34%	2.11%	-1.29%	8.99%
BRODMANN AREA 19 LEFT	8.93%	1.87%	-8.90%	-3.97%	2.04%	-9.61%	7.43%	2.85%	-11.64%	9.97%	-1.89%	11.44%	-8.82%	-9.30%	-2.89%	-12.52%	-6.46%	1.03%	-6.78%	2.52%	-6.78%	5.59%	0.91%	0.84%	7.42%	0.03%	5.84%	2.47%	-10.59%	-4.50%	-2.97%	-1.18%	6.84%
BRODMANN AREA 19 RIGHT	8.09%	-3.65%	-4.91%	-6.42%	5.86%	-11.03%	1.65%	2.31%	-16.74%	8.67%	-4.43%	10.05%	-12.02%	-5.87%	-0.82%	-13.34%	-9.91%	3.78%	-9.96%	-4.09%	-9.96%	2.33%	3.18%	-8.48%	-2.27%	-2.14%	7.29%	1.79%	-4.28%	-4.18%	2.65%	-2.48%	7.01%
BRODMANN AREA 20 LEFT	7.55%	2.15%	3.47%	-0.56%	22.68%	-6.71%	7.34%	6.98%	-18.90%	2.61%	8.22%	5.93%	-9.09%	-5.30%	6.73%	-11.09%	-2.75%	7.64%	-5.19%	6.13%	-5.19%	-7.56%	-4.68%	-13.55%	5.38%	3.96%	15.51%	6.66%	2.50%	2.13%	7.97%	1.32%	8.66%
BRODMANN AREA 20 RIGHT	8.65%	10.90%	-5.89%	-10.38%	21.09%	-5.82%	4.76%	-8.24%	-18.31%	1.67%	7.40%	8.85%	-8.17%	9.12%	7.40%	-16.77%	-8.27%	10.84%	2.14%	13.16%	2.14%	-9.54%	-4.67%	-0.22%	3.40%	-2.99%	-0.35%	3.56%	2.72%	1.34%	5.88%	0.82%	8.94%
BRODMANN AREA 21 LEFT	6.43%	10.60%	1.40%	-1.27%	15.07%	-3.48%	11.05%	-1.57%	-18.55%	4.67%	-5.65%	9.29%	-4.51%	-7.31%	0.92%	-9.67%	4.72%	4.29%	0.43%	4.93%	0.43%	-13.27%	-2.14%	-6.78%	-4.55%	7.52%	7.26%	1.54%	-5.59%	3.50%	3.88%	0.44%	7.42%
BRODMANN AREA 21 RIGHT	6.93%	6.97%	-5.61%	-3.49%	17.26%	-4.13%	3.81%	-6.93%	-21.45%	-2.98%	8.90%	15.12%	-6.50%	0.31%	10.64%	-12.29%	-9.28%	14.85%	-2.11%	10.63%	-2.11%	-7.93%	-9.95%	-0.89%	-1.42%	3.84%	4.72%	-2.72%	-3.88%	-0.36%	-1.23%	-0.04%	8.67%
BRODMANN AREA 22 LEFT	1.48%	3.76%	-0.73%	-3.98%	11.96%	-13.66%	8.85%	-0.15%	-26.20%	10.41%	5.13%	-3.00%	-6.62%	-3.91%	2.35%	-6.52%	-2.61%	7.23%	-5.67%	4.09%	-5.67%	-7.69%	-3.07%	-8.40%	-2.93%	3.47%	20.37%	5.07%	1.71%	-2.19%	8.76%	-0.27%	8.56%
BRODMANN AREA 22 RIGHT	19.05%	11.86%	-0.59%	-0.05%	1.09%	-6.58%	9.40%	-1.44%	-21.69%	2.35%	6.12%	5.66%	-12.77%	6.79%	5.11%	-8.28%	-0.26%	5.43%	-3.81%	6.56%	-3.81%	-3.16%	-2.81%	-4.33%	-1.38%	2.53%	8.93%	-3.33%	-0.14%	-12.81%	8.38%	0.39%	8.07%
BRODMANN AREA 23 LEFT	3.43%	-2.46%	-2.14%	-4.22%	4.31%	-10.77%	12.24%	7.26%	-20.45%	0.59%	-6.87%	7.40%	-7.06%	-3.02%	-2.48%	-0.69%	-2.80%	8.46%	-8.94%	-5.69%	-8.94%	-10.21%	-0.86%	-3.51%	7.12%	5.40%	6.87%	-4.21%	-12.49%	-11.21%	18.65%	-1.53%	8.27%
BRODMANN AREA 23 RIGHT	5.79%	-7.71%	-3.69%	-5.82%	4.98%	-4.73%	14.40%	6.18%	-16.48%	-2.40%	-0.61%	8.63%	-4.09%	-1.58%	2.91%	-3.51%	-6.27%	12.22%	-9.79%	-6.48%	-9.79%	-10.25%	-3.80%	-4.91%	7.05%	11.05%	8.97%	-2.87%	-9.69%	-9.57%	17.01%	-0.80%	8.38%
BRODMANN AREA 24 LEFT	15.03%	6.02%	-8.43%	0.63%	-0.57%	-3.08%	-2.74%	-3.86%	-28.73%	2.43%	7.06%	22.12%	4.49%	-0.99%	0.42%	-10.00%	-5.21%	4.33%	-10.68%	-3.95%	-10.68%	-4.26%	1.97%	4.03%	-4.28%	7.40%	-1.39%	-0.78%	0.50%	-2.56%	9.89%	-0.51%	8.87%
BRODMANN AREA 24 RIGHT	11.61%	6.41%	1.43%	0.52%	7.01%	-5.52%	4.80%	-0.40%	-26.45%	4.19%	4.11%	15.40%	-8.60%	0.99%	-0.22%	-4.34%	-5.46%	4.66%	-7.99%	-1.61%	-7.99%	-5.90%	5.94%	0.74%	-3.26%	4.72%	2.64%	1.99%	1.32%	1.76%	6.44%	0.29%	7.50%
BRODMANN AREA 25 LEFT	7.63%	-0.55%	7.39%	-5.31%	20.03%	-10.44%	0.69%	-14.93%	-21.88%	-8.19%	20.09%	14.37%	2.28%	5.86%	22.13%	-17.29%	0.17%	-0.49%	-2.83%	-1.12%	-2.83%	-8.05%	-2.60%	0.58%	-9.58%	7.23%	23.93%	11.29%	-6.11%	-9.63%	12.19%	1.10%	11.60%
BRODMANN AREA 25 RIGHT	5.95%	1.89%	4.99%	-5.40%	3.68%	-16.63%	9.67%	-5.32%	-18.60%	-4.92%	10.05%	11.13%	-11.49%	0.44%	5.81%	-14.96%	-4.97%	-0.58%	-0.52%	6.29%	-0.52%	-10.28%	-4.47%	7.94%	-4.42%	9.92%	19.10%	4.67%	4.18%	-9.40%	10.21%	0.11%	9.05%
BRODMANN AREA 28 LEFT	-0.35%	3.70%	1.58%	-4.38%	18.58%	-17.45%	-2.66%	2.92%	-25.61%	-1.20%	1.74%	2.30%	-10.15%	-3.37%	3.03%	-2.72%	3.68%	3.95%	-4.18%	0.80%	-4.18%	-2.52%	1.72%	0.50%	4.91%	4.67%	8.50%	4.61%	4.33%	-9.45%	-1.55%	-0.59%	7.77%
BRODMANN AREA 28 RIGHT	2.48%	-4.91%	0.93%	-7.52%	13.51%	-1.41%	-1.01%	-0.39%	-23.25%	-4.84%	9.80%	6.48%	-5.30%	6.90%	2.93%	-17.45%	-1.09%	12.39%	0.63%	-0.06%	0.63%	-3.32%	-10.19%	-0.32%	-3.33%	-2.52%	7.80%	2.67%	11.46%	-2.30%	2.60%	-0.26%	7.88%
BRODMANN AREA 31 LEFT	7.61%	-1.52%	-6.99%	-2.98%	17.01%	-12.24%	10.90%	-1.22%	-13.07%	-0.64%	-6.33%	5.59%	-1.54%	-8.37%	-2.70%	-12.81%	-7.09%	8.25%	-6.78%	-2.28%	-6.78%	-2.72%	-2.99%	-0.66%	2.99%	2.06%	11.91%	9.76%	-10.32%	-3.96%	0.43%	-1.21%	7.58%
BRODMANN AREA 31 RIGHT	14.20%	-1.69%	-8.83%	2.88%	8.13%	-11.39%	2.42%	0.95%	-11.77%	8.61%	-7.01%	3.75%	0.75%	-2.47%	-0.71%	-12.65%	-6.30%	13.47%	-5.13%	-6.99%	-5.13%	-4.21%	-8.38%	1.62%	0.00%	3.90%	15.73%	-0.11%	-11.44%	-3.67%	10.54%	-0.68%	7.86%
BRODMANN AREA 32 LEFT	3.97%	9.78%	-10.28%	-5.69%	12.71%	-12.17%	5.00%	-4.63%	-20.06%	-0.47%	0.82%	8.40%	-0.90%	-4.63%	-0.83%	-4.90%	-5.31%	2.36%	-6.70%	6.59%	-6.70%	-1.62%	-0.72%	6.02%	3.29%	7.22%	14.26%	-1.05%	-1.62%	0.15%	3.48%	-0.14%	7.34%
BRODMANN AREA 32 RIGHT	4.39%	7.47%	-4.50%	-3.18%	14.43%	-8.92%	3.60%	-0.75%	-19.73%	1.51%	2.56%	9.91%	-3.00%	-13.65%	4.57%	-7.31%	-3.47%	-1.38%	-9.39%	0.93%	-9.39%	-10.10%	-4.63%	4.34%	3.68%	15.88%	16.61%	3.74%	-4.40%	-4.84%	2.66%	-0.40%	8.38%
BRODMANN AREA 36 LEFT	-0.21%	-1.06%	-3.50%	1.16%	21.11%	-9.56%	8.44%	5.80%	-19.88%	0.20%	2.27%	5.75%	-9.08%	7.12%	7.80%	-0.23%	-2.01%	-0.81%	-4.32%	-0.26%	-4.32%	-11.22%	5.69%	-3.89%	0.28%	-4.45%	12.59%	-0.67%	2.13%	-12.12%	3.98%	-0.11%	7.84%
BRODMANN AREA 36 RIGHT	10.97%	-5.40%	4.97%	-7.30%	12.75%	-6.72%	11.58%	4.30%	-29.17%	-2.12%	6.24%	8.57%	-1.09%	14.06%	-2.63%	-20.65%	7.23%	15.88%	3.48%	6.27%	3.48%	0.24%	1.13%	-2.35%	15.36%	-1.05%	8.51%	2.71%	18.58%	-3.14%	6.33%	2.61%	10.10%
BRODMANN AREA 37 LEFT	-0.38%	-0.78%	-8.25%	-2.44%	10.86%	-3.90%	2.75%	-5.84%	-3.34%	6.50%	-1.83%	13.82%	-0.74%	-7.38%	-3.62%	-9.88%	6.36%	-0.14%	-11.70%	0.30%	-11.70%	10.13%	-2.92%	-10.12%	-3.39%	15.11%	10.85%	1.51%	-12.32%	-5.76%	-10.45%	-1.25%	7.74%
BRODMANN AREA 37 RIGHT	9.67%	-2.57%	-7.63%	-9.12%	7.19%	1.18%	4.57%	7.84%	-20.86%	8.01%	-7.67%	9.75%	-6.77%	-6.58%	-9.92%	-6.88%	-7.87%	-5.75%	6.09%	9.22%	6.09%	-3.46%	-9.59%	-4.47%	-3.87%	1.80%	4.55%	5.84%	-11.49%	3.65%	8.27%	-0.99%	7.96%
BRODMANN AREA 38 LEFT	6.40%	0.24%	2.56%	-8.16%	36.78%	-0.27%	6.05%	0.77%	-23.01%	4.93%	7.32%	-1.09%	-14.69%	0.30%	10.26%	-10.50%	-0.95%	6.54%	2.69%	8.47%	2.69%	-3.08%	-2.10%	-17.42%	-7.43%	-3.13%	5.49%	12.41%	1.83%	5.03%	4.38%	1.07%	10.41%
BRODMANN AREA 38 RIGHT	2.96%	16.57%	-5.99%	-6.87%	19.34%	-5.59%	6.75%	-8.52%	-18.94%	-1.90%	6.50%	14.82%	-14.81%	-2.75%	10.02%	-15.80%	-1.05%	12.16%	-0.66%	6.73%	-0.66%	-7.69%	-5.26%	1.24%	2.28%	-8.38%	6.99%	16.81%	-2.77%	-4.54%	3.93%	0.48%	9.69%
BRODMANN AREA 39 LEFT	3.51%	-6.36%	2.69%	-5.30%	11.03%	-3.87%	5.80%	1.35%	-19.57%	0.49%	-5.33%	7.47%	-2.98%	-12.22%	-3.45%	-14.64%	5.84%	10.57%	-4.88%	2.61%	-4.88%	-5.64%	-3.16%	-0.34%	3.14%	3.20%	20.61%	13.44%	-6.59%	-1.29%	-2.17%	-0.35%	8.22%
BRODMANN AREA 39 RIGHT	12.25%	-2.02%	-3.65%	-10.32%	5.81%	-12.31%	6.05%	4.55%	-15.54%	12.33%	-0.35%	11.69%	-4.14%	1.40%	-11.33%	-9.91%	-7.95%	15.94%	-8.15%	-8.16%	-8.15%	3.41%	-9.48%	-3.04%	-0.58%	17.12%	8.38%	5.67%	-9.47%	-12.40%	2.29%	-0.97%	9.19%
BRODMANN AREA 40 LEFT	-1.85%	-4.78%	-8.62%	-12.65%	12.23%	-7.86%	7.55%	-4.95%	-14.22%	2.34%	-0.17%	1.26%	-6.64%	-3.97%	-0.17%	-15.00%	4.04%	13.45%	-6.88%	10.30%	-6.88%	-11.54%	-3.61%	0.87%	2.16%	-2.65%	17.52%	-1.94%	-10.19%	-9.52%	8.84%	-1.73%	8.36%
BRODMANN AREA 40 RIGHT	21.55%	0.46%	-3.20%	-10.57%	3.09%	-14.15%	7.02%	-4.02%	-15.60%	10.48%	9.88%	0.80%	-9.37%	-3.14%	10.83%	-4.93%	-7.31%	8.01%	-11.84%	-5.82%	-11.84%	-5.06%	-5.93%	-2.98%	0.70%	13.40%	22.35%	-2.66%	-2.61%	-4.92%	-1.82%	-0.62%	9.60%
BRODMANN AREA 44 LEFT	-0.23%	4.97%	-3.25%	0.39%	14.24%	-9.12%	-1.87%	-4.60%	-17.01%	1.01%	10.93%	3.80%	-1.30%	-0.65%	7.65%	-10.02%	-0.14%	4.81%	-3.13%	2.72%	-3.13%	-6.28%	-10.03%	-1.04%	-5.58%	2.82%	18.18%	1.96%	-0.81%	-6.90%	8.22%	-0.11%	7.33%
BRODMANN AREA 44 RIGHT	8.59%	9.59%	-6.79%	-5.06%	21.41%	-8.97%	1.65%	5.48%	-30.08%	-1.06%	-2.00%	-1.31%	-0.07%	2.57%	1.44%	-7.17%	0.63%	2.75%	-4.88%	-1.37%	-4.88%	-11.82%	-7.17%	1.12%	7.29%	10.57%	14.48%	-5.69%	3.45%	-10.87%	6.74%	-0.37%	9.32%
BRODMANN AREA 45 LEFT	-2.98%	6.00%	-10.79%	7.29%	17.58%	-10.50%	0.92%	-6.02%	-17.53%	-3.33%	2.28%	0.27%	0.25%	-4.48%	2.72%	-8.06%	-0.21%	11.18%	-8.36%	1.56%	-8.36%	-12.52%	-4.99%	-8.18%	0.56%	9.96%	24.44%	6.41%	1.53%	-5.01%	6.21%	-0.39%	8.95%
BRODMANN AREA 45 RIGHT	3.69%	9.23%	-1.51%	-10.32%	17.17%	-9.41%	-3.86%	1.14%	-22.82%	-6.09%	0.08%	-0.14%	1.36%	12.64%	5.86%	-11.49%	3.84%	6.04%	-5.39%	-4.78%	-5.39%	-12.69%	1.26%	-8.15%	11.32%	13.49%	7.03%	4.66%	-3.32%	-4.43%	4.61%	-0.20%	8.73%
BRODMANN AREA 46 LEFT	-2.15%	1.16%	-3.18%	-4.14%	18.57%	-7.86%	7.86%	-5.51%	-18.12%	-1.54%	5.75%	3.03%	1.96%	-5.70%	12.09%	-9.68%	4.89%	17.41%	-5.93%	-0.77%	-5.93%	-13.98%	-4.88%	0.03%	-5.88%	21.97%	22.33%	3.05%	-7.27%	2.96%	6.62%	0.88%	9.84%
BRODMANN AREA 46 RIGHT	5.82%	5.77%	-10.68%	-3.05%	8.76%	-4.60%	6.75%	0.21%	-18.68%	-0.93%	10.44%	-0.43%	3.60%	2.29%	11.34%	-14.41%	-0.01%	8.30%	-6.59%	2.85%	-6.59%	-7.86%	-12.24%	-6.11%	3.12%	4.28%	19.71%	1.46%	-0.87%	6.75%	1.55%	0.32%	8.22%
BRODMANN AREA 47 LEFT	-9.85%	5.92%	-5.77%	-2.82%	13.48%	-2.73%	0.73%	-4.15%	-21.41%	1.01%	15.18%	3.90%	0.94%	3.28%	7.33%	-6.32%	-0.27%	12.05%	-6.26%	7.27%	-6.26%	-10.80%	-19.14%	-1.28%	-0.07%	13.00%	21.01%	1.17%	-3.26%	-7.04%	10.36%	0.30%	9.54%
BRODMANN AREA 47 RIGHT	2.67%	7.73%	-3.22%	-8.21%	11.21%	-8.89%	6.36%	0.12%	-17.26%	0.80%	3.37%	-2.50%	-1.13%	9.55%	13.38%	-8.47%	-4.96%	6.25%	-6.55%	9.05%	-6.55%	-4.74%	-9.00%	-3.99%	-1.36%	18.69%	-0.01%	-3.29%	0.28%	-7.48%	10.28%	0.07%	8.01%
