# Supplementary material for: Hyperbaric Oxygen Therapy Can Improve Post Concussion Syndrome Years after Mild Traumatic Brain Injury - Randomized Prospective Trial
Source: PLoS One. 2013 Nov 15;8(11):e79995. doi: 10.1371/journal.pone.0079995 (PMC3829860; doi:10.1371/journal.pone.0079995)
Supplement: Protocol S1 — Clinical study protocol. (DOCX) [file pone.0079995.s002.docx]

S1: Clinical Study Protocol

| Study Title: | Hyperbaric Oxygen Therapy can Improve Post Concussion Syndrome Years after Mild Traumatic Brain Injury - Randomized Prospective Trial |
| --- | --- |
|  |  |
|  |  |
|  |  |
| Protocol Number:  Investigational Product: | HBOT-TBI -01 Version 1.0    Hyperbaric Oxygen Therapy, 1.5ATM, 100% oxygen  Hyperbaric chamber, Multiplace at Assaf-Harofeh Medical Center, Israel |
|  |  |
| Primary Investigator:  Study Director: | Dr. Shai Efrati  Fax: +972-(0)8-920-4989  Phone: +972-(0)8-977-9393/5  Cell : +972-549-212-866  Email : [efratishai@013.net](mailto:efratishai@013.net)  Yair Bechor Fax: +972-(0)8-920-4989  Cell: +972-577-345-624 |
|  |  |
|  |  |

**Content**

**Pages**

Background………………………………………………………………………………....3-5

Objectives…………………………………………………………………………………....6

Study design……………………………………………………………………………......6-8

Inclusion criteria……………………………………………………………………..6

Exclusion criteria…………………………………………………………………..6-7

Study protocol……………………………………………………………………....7-8

End-point evaluation ……………………………………………………………………….8-11

Neurocognitive evaluation…………………………...…………………………….8-9
Brain functional Imaging……………………………………………………….…9-10

Quality of life ……………………………………………………………………..10-11

Administration and regulation……………………………………………………………...12

Informed Consent…………………………………………………………………..12

Confidentiality ……………………………………………………………………..12

Study Files……………………………………………………………………….…12

Statistical considerations...……………………………………………………………...13-14

Safety Analysis Set………………………………………………………….……..13

Primary Efficacy Analysis Set……………………………………………………..13

Sample Size Considerations………………………………………………….…....14

Primary Efficacy Analysis……………………………………………………….14-15

Randomization ……………………………………………………………………….…..15-16

Adverse Events……………………………………………………………………………16-19

References ………………………………………………………………………………..20-21

Appendix. EQ-5D questionnaire …………………………………………………………22-23

**Background**

Traumatic brain injuries (TBI) are a major cause of morbidity and mortality leading to major long term consequences on both a personal and national level with an estimated 5.3 million Americans suffering from permanent TBI related disabilities [[1](#_ENREF_1)]. Due to improvements in emergency medical care, transportation and specialized trauma facilities, the number of people surviving TBI with impairment has significantly increased in recent years. The long term consequences of TBI are vast, affecting a large number of people with a substantial effect on the patients themselves, their families and society as a whole. These injuries also impart a significant economic burden on those involved, both personally and as a society. Approximately 80,000 – 90,000 individuals suffer from long term disabilities annually [[2](#_ENREF_2)], and the estimated costs including both direct medical care and indirect expenses. Lost earning potential, for example, were over 60 million dollars in 2001 in the US [[3](#_ENREF_3)].

The long term sequel of TBI may include impairment of the individuals physical, cognitive and psychosocial functioning. The neurological consequences are numerous and complex affecting various sites and functions. Sensory, motor and autonomic systems may all be affected and symptoms may include headaches, seizures, visual defects, movement disorders and sleep disorders. The cognitive consequences likewise are broad and varied. Amongst the symptoms most commonly noted are memory deficits and attention and concentration deficits. There may be impaired executive function, problem solving abilities and language difficulties as well as problems with planning, information processing, judgment and insight. While some of these symptoms may be apparent immediately after the injury, others may present days, weeks or months after the initial trauma [[1](#_ENREF_1),[4](#_ENREF_4)]. Among all patients suffering from TBI, mild TBI (mTBI) patients are usually under-diagnosed, and early intervention and rehabilitation is often neglected due to lack of contusions or hemorrhage detectable by anatomical imaging at the acute stage of the injury [[5](#_ENREF_5)]. About 70–90% of all treated brain injuries are mild, and the incidence of hospital administrated patients with mild traumatic brain injury is estimated to be 100–300/100,000 population. However, most of the cases of mild TBI are not treated at hospitals, and the true population-based rate is probably above 600/100,000 [[6](#_ENREF_6)].

Single photon emission CT (SPECT) scans have been found to be effective in evaluating post traumatic lesions in mild TBI patients and are useful for follow up of recovery. In a prospective study to evaluate the affectivity of SPECT scans in the diagnosis of patients with mTBI and its correlation with common clinical symptoms such as post concussion syndrome (PCS), post traumatic amnesia (PTA) and loss of consciousness (LOC), Gawda et al. [[7](#_ENREF_7)] found perfusion abnormalities in 63% of the patients. This as opposed to positive CT findings in only 34% of these patients. Hence, SPECT scan was found to be more sensitive than CT [[7](#_ENREF_7)].

Hyperbaric Oxygen Therapy (HBOT) has been in use since the 1930's when it was initially used for decompression sickness. Shortly thereafter physicians started using HBOT for a variety of other conditions. The use of HBOT for neurological indications started in the early 1960's with the work by Smith et al showing its protective effect in cerebral ischemia and that of Saltzman showing effectiveness in stroke patients [[8](#_ENREF_8)]. HBOT is the inhalation of 100% oxygen at pressures exceeding 1 atmosphere absolute (ATA) in order to enhance the amount of oxygen dissolved in the blood and body fluids, thereby allowing for increased oxygen delivery to the tissues. Under normobaric conditions, the amount of oxygen dissolved in the blood is only 0.3 ml/dl. At 1.5 ATA this amount increases 10 fold to 3.2 ml/dl. Using an animal model of brain injured mice Daugherty et al. [[9](#_ENREF_9)] found a 250% increase in the local brain tissue oxygen levels between 100% oxygen administered at 1 ATA (103 mmHg) as opposed to that given at 1.5 ATA (247 mmHg). This seems to suggest that dissolved O2 is more readily available to the brain tissue than hemoglobin bound oxygen [[10](#_ENREF_10)]. Additionally, work by several investigators seems to indicate that HBOT allows for more efficient use of baseline oxygen levels by injured brain tissue following treatments, which in turn leads to a positive persistent affect on this tissue [[10](#_ENREF_10)].

The mechanism by which HBOT is thought to improve the outcome of brain injury is multifaceted, The Neubauer and Walker theory postulates that HBOT improves cerebral metabolism by improving functioning of the dormant neurons and stimulating axonal growth [[11](#_ENREF_11)]. Zhang et al suggest that potential targets of oxygen therapy include prevention of apoptosis, inhibition of neuroinflammation and BBB damage [[12](#_ENREF_12)] . There is stimulation of angiogenesis and neovascularization, as well as direct effects on blood vessels in the brain, and maintenance of BBB integrity [[10](#_ENREF_10),[13](#_ENREF_13)]. Similarly, SPECT scans have demonstrated a positive effect on the cerebral blood flow (CBF) in the damaged brain following HBOT [[10](#_ENREF_10),[11](#_ENREF_11),[14](#_ENREF_14),[15](#_ENREF_15),[16](#_ENREF_16)].

There are well documented animal models of TBI and a growing body of literature using HBOT on these animal models verifying the above stated hypotheses and findings. Sun et al demonstrated improved penumbral oxygenation following HBO treatment in focal ischemia using an animal model by measuring both extrinsic and intrinsic markers of hypoxia [[17](#_ENREF_17)]. Harch et al used a rat model of TBI to evaluate HBOT effects on spatial learning and memory, as well as its affect on blood vessel density [[18](#_ENREF_18)]. HBOT also brings about improved neurocognitive functioning in patients suffering from chronic severe brain injury [[10](#_ENREF_10),[19](#_ENREF_19),[20](#_ENREF_20),[21](#_ENREF_21),[22](#_ENREF_22)]. However, none of these human trials were prospective, randomized clinical trials in TBI patients.

**Objectives**

The aim of the current study is to evaluate, for the first time in a prospective cross-over, randomized study, the effect of hyperbaric oxygen therapy (HBOT) on patients with chronic neurocognitive deficiency due to mild TBI.

- The primary endpoints of the study are to evaluate the effects of the HBOT on:
- Neurocognitive function (using Mindstreams neurocognitive battery)
- Brain metabolism (as visualized by SPECT)
- The secondary endpoints included quality of life evaluation.

The safety of the HBOT in this post TBI population will also be evaluated and any adverse event will be recorded.

**Study design**

A prospective, randomized, controlled, cross-over trial.

The study will be executed in the hyperbaric chamber and in the research and development unit of Assaf Harofeh Medical Center, Israel.

The brain SPECT evaluation will be executed in Nuclear Medicine institute in Assaf-Harofeh Medical Center, Israel.

**Inclusion criteria**

- An incident of mild traumatic brain injury 1-5 years prior to the inclusion in the study.
- All patients have to have complaints regarding persistent (stable not improving) neurocognitive deficiencies (memory, concentration, etc.) without noticeable improvement during the last month prior to their enrolment.
- Age 18 years or older.

**Exclusion criteria**

Patients will be excluded if they will have one of the following criteria:

- Dynamic neurologic improvement or worsening during the past month;
- Had been treated with HBOT for any other reason prior to their inclusion;
- Have any other indication for HBOT;
- Chest pathology incompatible with pressure changes;
- Inner ear disease;
- Patients suffering from claustrophobia;
- Inability to sign written informed consent;
- Smoking patients will not be allowed to smoke during the study and if they would not comply with this demand they will be excluded.

***Study protocol***

After signing the informed consent, patients will be randomized in 1:1 manner into the treated or the control-cross group. After the randomization, patients will be invited for baseline evaluation that will include full review of their medical status and complete physical examination. All patients will present baseline anatomical scan (CT/MRI). After their inclusion, patients will be randomized to two groups: a treated group and a cross group. All patients will go through evaluation of their neurocognitive function using neurocognitive testing battery, quality of life using EQ-5D questionnaire and brain functional imaging (rCBF-SPECT scan). The patients in the treated group will be evaluated twice – at baseline and after 2 months of HBOT treatment. The patients of the cross group will be evaluated three times – at baseline, after a 2 month control period of no treatment and after a consequent 2 month period of HBOT treatment. We emphasize that the study will be a cross-over trial and patients in the cross group will receive HBOT treatment after their second evaluation and will go through a third neurological evaluation after the cross, when they will complete their HBOT.

The following HBOT treatment protocol will be practiced: The patients will go through 40 HBO treatments (each treatment session will be given on a separate day), distributed over two months (five days a week). Each session will be for 60 minutes in 100% oxygen atmosphere and at pressure of 1.5 ATA.

***End-point evaluation***

**Neurocognitive evaluation**

The neurocognitive evaluation will be done at baseline and after 2 months for all patients. In the cross group a 3^rd^ evaluation will be performed after 4 month- after crossing and completing 2 months of HBOT treatment. Cognitive function will be assessed using the one-hour Mindstreams Computerized Cognitive Test Battery (Mindstreams; NeuroTrax Corp., NY). Detailed description of the tests can be found on Neurotrax website ([www.neurotrax.com](http://www.neurotrax.com)).

To minimize differences in age and education, each outcome parameter will be normalized and fit to an IQ-like scale (mean=100, S.D=15) according to patient's age and education. Normalized subsets of outcome parameters will be aggregated to produce four index scores relevant to mild TBI: **Memory, Attention, Executive function,** and **Information Processing Speed**.

Three different test versions exist in the Mindstreams testing battery to allow repeated administrations, test-retest reliability for those versions were evaluated and found high, with no significant learning effect [[23](#_ENREF_23),[24](#_ENREF_24)].

**Brain functional Imaging - SPECT imaging and Analysis**

Single photon emission computed tomography (SPECT) will be conducted before and after HBOT. Subjects will lay supine in a quiet dimly lit room for 20 min prior to injection of the radiopharmaceutical. Apart from administration of the injection by a physician, they will remain alone in the room during this period. Subjects will be asked to remain at rest for 10 min after the injection of the radiopharmaceutical to allow uptake of the radiopharmaceutical in the brain.

An injection of 925-1110 MBq (25-30 mCi) of technetium-99m ethyl cysteinate dimmer (Tc-99m-ECD) will be given into an arm vein through a previously placed intravenous cannula. SPECT imaging of the brain will be performed, at 40-60 min post injection, with the subject’s head supported by a headrest, using a dual detector gamma camera (ECAM or Symbia T, Siemens Medical Systems) equipped with high resolution collimators. Data will be acquired in the step-and-shoot mode, using a 360 degree circular orbit, with the detectors of the gamma camera as close as possible to the subject’s head. The camera used for imaging will be noted for each subject and the same camera will be used for the follow-up study. Data will be acquired using a 128׳128 image matrix in 3 degree steps of 20 seconds per step. Data will be reconstructed by iterative reconstruction with no filtering. The Chang method (*μ*=0.12/ cm) will be used for attenuation correction.

Intra subject visual analysis will be conducted using a process in which pre and post treatment studies are fused and normalized to pre-treatment whole brain activity.

Visual analysis will be carried out by two nuclear medicine physicians who compared the scans independently and grad them as either: 1=no change, 2=mild change and 3=significant change. Where, no change is assign when no visual difference is noted in the number or size of perfusion deficits, mild change is given when a reduced number of perfusion defects are noted or the size of the perfusion defects is reduced. Significant change should be attributed when a global perfusion increment is observed in addition to diminution of defect numbers or size. Differences in evaluation will be resolve after reviewing the images together. Scan visual interpretation will be carried out while blinded to any laboratory or clinical data.

A comparison of the SPECT results with anatomical imaging (CT or MRI) will be done in order to evaluate the extent of perfusion deficit in relation to the anatomical lesion.

**Quality of life evaluation**

Quality of live will be evaluated by the EQ-5D questionnaire [[25](#_ENREF_25),[26](#_ENREF_26)]. EQ-5D essentially consists of two pages - the EQ-5D descriptive system and the EQ visual analogue scale (EQ VAS). The EQ-5D descriptive system comprises the following 5 dimensions: mobility, self-care, usual activities, pain/discomfort and anxiety/depression. Each dimension has 3 levels: no problems, some problems, extreme problems.

The EQ VAS records the respondent’s self-rated health on a vertical, visual analogue scale where the endpoints are labeled “Best imaginable health state” and “Worst imaginable health state” (0 is denoting the worst imaginable health state while 100 is denoting the best imaginable health state). The validity and reliability of the EQ­5D questionnaire have been tested [[27](#_ENREF_27),[28](#_ENREF_28)].

The EQ-5D questionnaire is attached in the appendix.

**Administration and regulation**

**Informed Consent**

The investigator will obtain written informed consent from the patient participating in this study after adequate explanation of the aims, methods, objectives, and potential hazards of the study and prior to undertaking any study-related procedures. The investigator must utilize a consent form for documenting written informed consent. Informed consent will be appropriately signed and dated by the patient or the subject’s legally authorized representative and the person obtaining consent.

**Confidentiality**

Subjects’ anonymity will be strictly maintained and their identities will remain protected from unauthorized parties. The information is not to be disclosed to any third party (except for medical stuff or employees or agents directly involved in the conduct of the study or as required by law).

**Study Files**

The medical records will be maintained adequately to enable good data storage and latter on management. Subjects' clinical source documents will include (but not limited to) the following: subject's hospital/clinic/ hyperbaric unit records, physician’s and nurse’s notes, appointment book, original laboratory reports, electroencephalogram (EEG), X-ray, SPECTs, CT and special assessment reports, consultant letters, screening and enrollment log, etc.

**Statistical Considerations**

## Analysis Sets

### Safety Analysis Set

The safety analysis set will consist of all subjects with whom the study treatment was initiated.

### Primary Efficacy Analysis Set

The primary endpoint parameter: *improvement in neurocognitive tests scores* will be measured following HBOT treatment for post TBI patients suffering from chronic neurocognitive deficiencies.

The primary efficacy analysis evaluation will include all subjects who completed the HBOT treatment or the control period, had no major protocol violations, and for whom there is a valid *mindstreams score* at baseline and following 2 months of HBOT treatment.

**Sample Size Considerations**

Presentation of sample size is based on achieving 80% power overall to demonstrate that improvement rate in *mindstreams score* is at least 12%. An improvement rate of at least 10% in the neurocognitive score in treatment group (compared to baseline, prior to HBOT treatment) would be an appropriate clinical target for HBOT treatment success and of sufficient interest to encourage further investigation of HBOT treatment for post TBI patients suffering chronic neurocognitive deficiencies.

Sample size is based on the assumption that exposure to the *mindstreams* tests at baseline without any additional training might induce 2% score improvement in the second test, following treatment, (test-retest effect). Assuming a true success rate of 12% a sample of N = 31 will provide 80% power to show that HBOT treatment induces at least 10% improvement on *neurocognitive test score*. This is based on a power analysis using the normal approximation for the binomial, with one-sided Alpha=0.05.

## Statistical Analysis

### Safety Analysis

Adverse events will be tabulated by treatment group, severity and relation to treatment.

### Primary Efficacy Analysis

For the primary end point, Neurocognitive function (using Mindstreams neurocognitive battery test score), we will compute Overall Proportion of Success:

$$\text{P(success) }=\text{ }\frac{\text{Total \# of Successes}}{\text{Total \# in Cohort}}$$

The following hypotheses are specified and will be tested separately:

For Neurocognitive *tests score* proportion of improvement:

H_0_: p_1=_ p _2_

H_1_: p_1>_ p_2_

While p1= Neurocognitive function (using Mindstreams neurocognitive battery *tests score*) improvement as a result of the HBOT training (assumed to be 0.12); and

p2= Neurocognitive function (using Mindstreams neurocognitive battery *tests score*) improvement as a result of test-retest effect (assumed to be 0.02)

This hypothesis will be tested by:

1. Constructing a one-sided, lower 95% confidence interval about the observed Overall Proportion of Success in the relevant cohort.
2. Examining whether the lower limit of the confidence interval is at or above the success criterion

Study success will be declared if the following is met:

1. Lower confidence limit of HBOT treatment success is at 0.12 or above

**Randomization**

Since the diversity of the patients included in the study, after signing the informed consent is expected to be high, no stratifies criteria will be used. Patients will be randomized in 1:1 fashion to either treatment or controlled-cross group.

There will be no placebo in the cross group during the control period. The only way to give “placebo” of HBOT, is to bring the patients to the hyperbaric chamber and to increase the environmental pressure in a way the patients will “feel the hyperbaric pressure” in their ears. However, Henry’s law states: “the amount of a given gas dissolved in a given type and volume of liquid is directly proportional to the pressure of that gas in equilibrium with that liquid”. Thus, hyperbaric environment significantly increases the dissolved oxygen pressure even if a person holding his breath [[29](#_ENREF_29)]. Moreover, the alternative of hosting the patients in the hyperbaric chamber without any pressure increase cannot serve as a real placebo since the patients will not feel the pressure. Accordingly, the only real “placebo” could be achieved by increasing the pressure and reducing the percentages of the oxygen but that holds significant safety consideration.

From the ethical point of view, it is deemed unethical to “treat” the patients (40 sessions in the hyperbaric chamber) while they actually know that it is a placebo. In order to somewhat compensate for this limitation, the control will be a cross group and after the 2^nd^ evaluation at the end of a control period they will be crossed to HBOT. The cross for treatment enables intra-group efficacy evaluation in both treatment and control group.

Since the patients will know they are not receiving HBOT during the control period they will not be blinded with regard to the treatment arm. Accordingly, the quality of life evaluations will not be blinded. However, the complete blindness evaluation can be assured in both of the primary end points of this study, The Mindstreams computerized evaluation and the brain SPECT evaluation done by the radiologist.

**Adverse Events**

An adverse event (AE) is any untoward medical occurrence in a clinical investigation subject administered a medicinal product and which does not necessarily have a causal relationship with this treatment. An AE can therefore be any unfavorable and unintended sign, symptom, or disease temporally associated with the use of a medicinal product, whether or not considered related to the medicinal product. Pre-existing events, which increase in severity or change in nature during or as a consequence of use of a medicinal product in human clinical trials, will also be considered AEs.

Any medical condition or clinically significant laboratory abnormality with an onset date before the screening visit and not related to study procedures is considered to be pre-existing, and should be documented in the case report form.

Any AE (i.e., a new event or an exacerbation of a pre-existing condition) with an onset date after the screening visit up to the last day on study (including the follow-up, off study medication period of the study), should be recorded as an AE on the appropriate CRF page(s).

An AE does not include:

- Medical or surgical procedures (e.g. surgery, Endoscopy, tooth extraction, transfusion); the condition that leads to the procedure are an adverse event.
- Pre-existing diseases or conditions or laboratory abnormalities present or detected prior to the screening visit that does not worsen.
- Situations where an untoward medical occurrence has not occurred (e.g. hospitalization for elective surgery, social and/or convenience admissions).
- Overdose of either study drug or concomitant medication without any signs or symptoms unless the subject is hospitalized for observation.

***Assessment of Adverse Events***

All AEs will be assessed by the investigator and recorded on the appropriate CRF page, including the date of onset and resolution, severity, relationship to study drug or study procedures, outcome and action taken with study medication.

The relationship to study drug therapy or study procedures should be assessed using the following definitions:

**No**: Evidence exists that the adverse event has an etiology other than the study drug or study procedures (e.g. pre-existing condition, underlying disease, intercurrent illness, or concomitant medication).

**Yes**: A temporal relationship exists between the event onset and administration of the study drug or between the event and the study procedures. It cannot be readily explained by the subject’s clinical state or concomitant therapies and, in the case of the study drug, appears with some degree of certainty to be related based on the known therapeutic and pharmacologic actions or adverse event profile of the study drug. In case of cessation or reduction of the dose, the event abates or resolves and reappears upon re-challenge. It should be emphasized that ineffective treatment should not be considered as causally related in the context of adverse event reporting.

These criteria in addition to good clinical judgment should be used as a guide for determining the causal assessment. If it is felt that the event is not related to study drug therapy, then an alternative explanation should be provided.

***Serious Adverse Events***

**A** **serious adverse event** (SAE) is defined as follows:

Any adverse drug experience occurring at any dose that results in any of the following outcomes:

- Death;
- Life-threatening situation (subject is at **immediate** risk of death);
- In-patient hospitalization or prolongation of existing hospitalization (excluding those for study therapy or placement of an indwelling catheter, unless associated with other serious events);
- Persistent or significant disability/incapacity;
- Congenital anomaly/birth defect in the offspring of a subject who received study drug;

Other: medically significant events that may not result in death, be immediately life-threatening, or require hospitalization, may be considered a SAE when, based upon appropriate medical judgment, they may jeopardize the Subject and may require medical or surgical intervention to prevent one of the outcomes listed in this definition.

Examples of such events are:

- Intensive treatment in an emergency room
- Blood dyscrasias or convulsions that do not result in hospitalization
- Development of drug dependency or drug abuse

The investigator should notify the Institutional Review Board (IRB) or Independent Ethics Committee (IEC) as soon as is practical, of serious events in writing where this is required by local regulatory authorities, and in accordance with the local institutional policy.

**References**

1. Control NCfIPa (2003) Report to Congress on Mild Traumatic Brain Injury in the United States: Steps to Prevent a Serious Public Health Problem. Atlanta, GA: Centers for Disease Control and Prevention.

2. Langlois JA, Rutland-Brown, W., Thomas, K.E. (2003) Traumatic brain injury in the United States: emergency department visits, hospitalizations, and deaths. In: Centers for Disease Control and Prevention NCfIPaC, editor. Atlanta, GA.

3. Finkelstein E, Corso, P., Miller, T., et al. (2006) The Incidence and Economic Burden of Injuries in the United States. NY: Oxford University Press.

4. Kushner D (1998) Mild traumatic brain injury: toward understanding manifestations and treatment. Archives of internal medicine 158: 1617-1624.

5. (1999) Consensus conference. Rehabilitation of persons with traumatic brain injury. NIH Consensus Development Panel on Rehabilitation of Persons With Traumatic Brain Injury. JAMA : the journal of the American Medical Association 282: 974-983.

6. Cassidy JD, Carroll LJ, Peloso PM, Borg J, von Holst H, et al. (2004) Incidence, risk factors and prevention of mild traumatic brain injury: results of the WHO Collaborating Centre Task Force on Mild Traumatic Brain Injury. Journal of rehabilitation medicine : official journal of the UEMS European Board of Physical and Rehabilitation Medicine: 28-60.

7. Gowda NK, Agrawal D, Bal C, Chandrashekar N, Tripati M, et al. (2006) Technetium Tc-99m ethyl cysteinate dimer brain single-photon emission CT in mild traumatic brain injury: a prospective study. AJNR American journal of neuroradiology 27: 447-451.

8. Jain KK (1996) Textbook Of Hyperbaric Medicine: Hogrefe & Huber.

9. Daugherty WP, Levasseur JE, Sun D, Rockswold GL, Bullock MR (2004) Effects of hyperbaric oxygen therapy on cerebral oxygenation and mitochondrial function following moderate lateral fluid-percussion injury in rats. Journal of neurosurgery 101: 499-504.

10. Rockswold SB, Rockswold GL, Defillo A (2007) Hyperbaric oxygen in traumatic brain injury. Neurological research 29: 162-172.

11. Golden ZL, Neubauer R, Golden CJ, Greene L, Marsh J, et al. (2002) Improvement in cerebral metabolism in chronic brain injury after hyperbaric oxygen therapy. The International journal of neuroscience 112: 119-131.

12. Zhang JH, Lo T, Mychaskiw G, Colohan A (2005) Mechanisms of hyperbaric oxygen and neuroprotection in stroke. Pathophysiology : the official journal of the International Society for Pathophysiology / ISP 12: 63-77.

13. Al-Waili NS, Butler GJ, Beale J, Abdullah MS, Hamilton RW, et al. (2005) Hyperbaric oxygen in the treatment of patients with cerebral stroke, brain trauma, and neurologic disease. Advances in therapy 22: 659-678.

14. Barrett KF, Masel B, Patterson J, Scheibel RS, Corson KP, et al. (2004) Regional CBF in chronic stable TBI treated with hyperbaric oxygen. Undersea & hyperbaric medicine : journal of the Undersea and Hyperbaric Medical Society, Inc 31: 395-406.

15. Neubauer RA, James P (1998) Cerebral oxygenation and the recoverable brain. Neurological research 20 Suppl 1: S33-36.

16. Neubauer RA, Gottlieb SF, Kagan RL (1990) Enhancing "idling" neurons. Lancet 335: 542.

17. Sun L, Marti HH, Veltkamp R (2008) Hyperbaric oxygen reduces tissue hypoxia and hypoxia-inducible factor-1 alpha expression in focal cerebral ischemia. Stroke; a journal of cerebral circulation 39: 1000-1006.

18. Harch PG, Kriedt C, Van Meter KW, Sutherland RJ (2007) Hyperbaric oxygen therapy improves spatial learning and memory in a rat model of chronic traumatic brain injury. Brain research 1174: 120-129.

19. Golden Z, Golden CJ, Neubauer RA (2006) Improving neuropsychological function after chronic brain injury with hyperbaric oxygen. Disability and rehabilitation 28: 1379-1386.

20. Hardy P, Johnston KM, De Beaumont L, Montgomery DL, Lecomte JM, et al. (2007) Pilot case study of the therapeutic potential of hyperbaric oxygen therapy on chronic brain injury. Journal of the neurological sciences 253: 94-105.

21. McDonagh M, Helfand M, Carson S, Russman BS (2004) Hyperbaric oxygen therapy for traumatic brain injury: a systematic review of the evidence. Archives of physical medicine and rehabilitation 85: 1198-1204.

22. Shi XY, Tang ZQ, Sun D, He XJ (2006) Evaluation of hyperbaric oxygen treatment of neuropsychiatric disorders following traumatic brain injury. Chinese medical journal 119: 1978-1982.

23. Schweiger A, Doniger, G.M., Dwolatzky, T., Jaffe, D., & Simon, E.S. (2003) Reliability of a novel computerized neuropsychological battery for mild cognitive impairment. Acta Neuropsychologica 1: 407-416.

24. Melton JL (2005) Psychometric evaluation of the Mindstreams neuropsychological screening tool. Panama City (FL): Navy Experimental Diving Unit (US). NEDU TR NEDU TR. 06-10 p.

25. Bretthauer M (2008) [Statistical significance and clinical relevance]. Tidsskrift for den Norske laegeforening : tidsskrift for praktisk medicin, ny raekke 128: 279.

26. (1990) EuroQol--a new facility for the measurement of health-related quality of life. The EuroQol Group. Health Policy 16: 199-208.

27. Brazier J, Jones N, Kind P (1993) Testing the validity of the Euroqol and comparing it with the SF-36 health survey questionnaire. Qual Life Res 2: 169-180.

28. van Agt HM, Essink-Bot ML, Krabbe PF, Bonsel GJ (1994) Test-retest reliability of health state valuations collected with the EuroQol questionnaire. Soc Sci Med 39: 1537-1544.

29. Muth CM, Radermacher P, Pittner A, Steinacker J, Schabana R, et al. (2003) Arterial blood gases during diving in elite apnea divers. International journal of sports medicine 24: 104-107.

**Appendix.** EQ-5D questionnaire
