# Supplementary material for: Hyperbaric Oxygen Therapy Can Improve Post Concussion Syndrome Years after Mild Traumatic Brain Injury - Randomized Prospective Trial
Source: PLoS One. 2013 Nov 15;8(11):e79995. doi: 10.1371/journal.pone.0079995 (PMC3829860; doi:10.1371/journal.pone.0079995)
Supplement: Text S1 — Crossover approach and simulated randomization. (DOC) [file pone.0079995.s005.doc]

**Text S5. Crossover approach and simulated randomization**

**Avantageuse:** Crossover trials are increasingly used in clinical research. The approach is utilized to compare the effectiveness of two or more treatments or compare treatment to sham (placebo) control, as is the case here. The patients are randomized into two groups. The patients in one group (the treated group) first undergo a period of treatment and then a control period. The patients in the other group (the control group) first undergo a period of control and then a period of treatment. The key advantage of the crossover approach vs. parallel group trials is that, by comparing the effects of treatment and sham control for each patient separately, the inherent statistical variations associated with patient-to-patient diversity are reduced.

**Concerns:** Crossover studies are prone to two major areas of bias, one associated with changes over time and the other with possible carryover effects. First, the time delay between treatments of the two groups can affect the results. For example, the treatment in the control group can appear less effective due to further progression of the disease during the control period. It is also possible that gaining experience and other time related effects on the individual subjects may interfere with the effects of intervention. For example, in the current study, more practice in performing the cognitive tests can affect the results. Regarding the carryover effects, residual carryover effects from the earlier treatment may affect the results of the consequent period. Therefore, a careful design of “wash out” period of the treatment effects between crossover trials is important.

In view of the above, and based on the experience of long lasting HBOT effects in mTBI (months), the patients of the treated group in the current study did not go through a two month control period following the treatment period.

The special nature of the crossover trials calls for special analyses to exploit the fact that each patient acts as her or his own control. To meet the challenge, we studied the individual changes in the cognitive indices and quality of life measures.

**Simulated randomization:** In the current study, there is an asymmetry between the two groups: First, while the patients of the control-cross group went through three evaluations (at baseline, after control and post HBOT), the patients of the treatment group went through two evaluations (at baseline and post HBOT). Second, while the two groups started with the same number of patients, the final number of patients in the control group was smaller (24 vs. 32) due to dropouts. In addition, as expected, there is a high patient-to-patient variability in the relative changes of the cognitive indices (see Fig. SI 5-1).


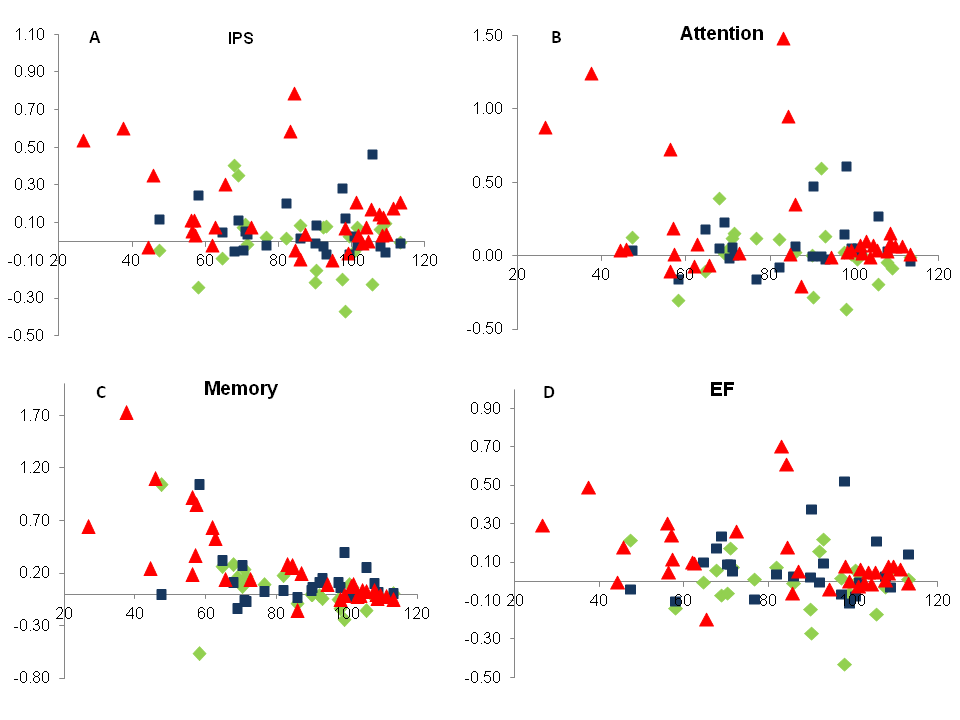


**Figure S1:** Illustration of the patient-to-patient variability in the individual relative changes in the cognitive indices. In this figure we show the relative changes in all four cognitive indices - Information Processing Speed (A), Attention (B), memory (C) and Executive functions (D) - for all the patients as a function of the baseline value of the memory score. The color code is: Red for the treated group, green after control period of the control group and blue after HBOT period of the control group.

It is transparent in the figure that some patients exhibited non-vanishing positive changes and others non-vanishing negative changes during the control period. In addition, few patients exhibited small negative changes in some of the cognitive indices also after HBOT periods.

Considering the above, it is important to rule out that the averaged observed improvements are an artifact or coincidental. To inspect the issue, we performed two types of “simulated randomizations” – shuffling of the individual tests and shuffling of the patients.

Shuffling of the individual tests is done as follows: 1. For each patient in the treated group, decide randomly (with probability 1/2) if the baseline scores are his/her true baseline scores or the post HBOT scores. Then, calculate for the patient the relative change in the general score according to the new “tests” and define it as the simulated score. 2. For each patient in the cross group, decide with probability 1/3 if the baseline scores are the original ones, the ones after control or the ones after HBOT. Then, for the scores that were not selected as baseline, decide with probability 1/2 which will be the ones after control and which after HBOT. Then calculate for the patient the simulated relative change in the general score for the control period and the HBOT period.

Shuffling of the patients was done as follows: We consider the ensemble of the 80 results of the relative changes of all the patients in the two groups – 32 results for the change following HBOT of the 32 patients of the treatment group, 24 results for the changes following control of the 24 patients of the control group, and 24 results for the changes following HBOT of the 24 patients of the control group. Next, we select randomly 24 changes and assign them to be the simulated results of the control group during control. Next we randomly select 24 changes and assign them to be the simulated results for the HBOT period of the control group. The left 32 changes are assigned to be the results for the HBOT of the treated group. We performed ***100,000 realizations*** of such combined shuffling and found that the likelyhood to obtain results similar to the obeserved ones (above 8% improvements following HBOT and less than 2% improvement following control) is very low; ***the probablity is smaller than 2x10^-4^***. Even if we retain the original relative changes (which introduces significant bias) and perform only patients shuffling, ***the probability*** ***is smaller than 0.02***.
